# Integrating stakeholders’ perspectives and spatial modelling to develop scenarios of future land use and land cover change in northern Tanzania

**DOI:** 10.1371/journal.pone.0245516

**Published:** 2021-02-12

**Authors:** Rebecca W. Kariuki, Linus K. Munishi, Colin J. Courtney-Mustaphi, Claudia Capitani, Anna Shoemaker, Paul J. Lane, Rob Marchant

**Affiliations:** 1 School of Life Sciences and Bio-Engineering, Nelson Mandela—African Institution of Science and Technology, Tengeru, Arusha, Tanzania; 2 Department of Environment and Geography, York Institute for Tropical Ecosystems, University of York, Heslington, York, North Yorkshire, United Kingdom; 3 Department of Archaeology and Ancient History, Uppsala University, Uppsala, Sweden; 4 Department of Archaeology, University of Cambridge, Cambridge, United Kingdom; 5 School of Geography, Archaeology and Environmental Science, University of the Witwatersrand, Johannesburg, South Africa; United Nations University Institute for Natural Resources in Africa, GHANA

## Abstract

Rapid rates of land use and land cover change (LULCC) in eastern Africa and limited instances of genuinely equal partnerships involving scientists, communities and decision makers challenge the development of robust pathways toward future environmental and socioeconomic sustainability. We use a participatory modelling tool, Kesho, to assess the biophysical, socioeconomic, cultural and governance factors that influenced past (1959–1999) and present (2000–2018) LULCC in northern Tanzania and to simulate four scenarios of land cover change to the year 2030. Simulations of the scenarios used spatial modelling to integrate stakeholders’ perceptions of future environmental change with social and environmental data on recent trends in LULCC. From stakeholders’ perspectives, between 1959 and 2018, LULCC was influenced by climate variability, availability of natural resources, agriculture expansion, urbanization, tourism growth and legislation governing land access and natural resource management. Among other socio-environmental-political LULCC drivers, the stakeholders envisioned that from 2018 to 2030 LULCC will largely be influenced by land health, natural and economic capital, and political will in implementing land use plans and policies. The projected scenarios suggest that by 2030 agricultural land will have expanded by 8–20% under different scenarios and herbaceous vegetation and forest land cover will be reduced by 2.5–5% and 10–19% respectively. Stakeholder discussions further identified desirable futures in 2030 as those with improved infrastructure, restored degraded landscapes, effective wildlife conservation, and better farming techniques. The undesirable futures in 2030 were those characterized by land degradation, poverty, and cultural loss. Insights from our work identify the implications of future LULCC scenarios on wildlife and cultural conservation and in meeting the Sustainable Development Goals (SDGs) and targets by 2030. The Kesho approach capitalizes on knowledge exchanges among diverse stakeholders, and in the process promotes social learning, provides a sense of ownership of outputs generated, democratizes scientific understanding, and improves the quality and relevance of the outputs.

## 1. Introduction

Land-use systems are shaped by many factors operating at multiple scales. These include interactions between humans and environmental processes [1,2]; global and regional environmental and market patterns [3,4]; historical legacies [5]; institutions, technologies and cultural practices that influence land uses [6,7]; and feedback among drivers and impacts of land use and land cover change (LULCC). LULCC is a cause and consequence of social-ecological processes because humans drive land use decisions at local to national scales, and in turn LULCC has consequences for climate change and its impacts, ecosystem service provisioning, and environmental degradation [2–4,6]. LULCC is also a product of management interventions such as reforestation, wildlife conservation, erosion control, and soil restoration, which prompt further land use decisions and responses. Disruptions of ecosystem services caused by LULCC indicate the need for incorporating LULCC in addressing sustainability challenges in land management, climate change adaptation, food security, and biodiversity and cultural loss [7,8].

This study examines these intersecting issues with reference to an area of the wider Serengeti ecosystem, northern Tanzania. Northern Tanzania is characterized by a mosaic of landscapes ranging from volcanic highlands in the northwest, montane forests in the northeast, agricultural areas on moist highland slopes, and wetlands, savannas, and grasslands throughout. The area contains biodiversity hotspots [9,10], has high geological diversity [11,12], several key paleoanthropological and archaeological sites [13,14], and supports a wide range of socioeconomic activities. The combination of natural resources, competing land uses, and a diversity of resource users requires several and often overlapping management priorities, including multiple types of Protected Areas, and local-to-global scales of connection and interaction. The Protected Areas provide significant revenue for Tanzania through wildlife conservation and tourism. For instance, in 2013, tourism from wildlife conservation contributed 9.9% of Tanzania’s Gross Domestic Product (GDP) and provided direct employment to >400,000 people [15].

Protected Areas face several management challenges such as habitat fragmentation, over exploitation of natural resources, climate change impacts, management ineffectiveness, and biodiversity loss [16–18]. In part, these challenges have their origins in the original setting of the Protected Areas that did not consider wildlife and cultural heritage as ‘resources’ for socioeconomic development [14]. Meeting the conservation objectives of Protected Areas now more than ever requires connectivity across space so that they are not operating in isolation [17,19,20]. Increasingly, novel solutions for sustaining biodiversity inside and outside of Protected Areas, that involve all sectors of society are needed [21,22]. The Man and Biosphere (MAB) and the Geoparks Programs of the United Nations Educational, Scientific and Cultural Organization (UNESCO) have been proposed as potential solutions for addressing the disconnect between biodiversity conservation and sustainable development [23,24]. The programs promote stakeholder awareness, communication, and participation in decision making for Biosphere Reserve management [25]. However, as successful management of Protected Areas requires addressing the insecurity and uncertainty of land tenure in community areas [20,26], maintaining sustainable biodiversity in places adjacent to Protected Areas continues to be challenged by ‘business as usual’ forms of agricultural expansion and increasing human population pressures [27–30].

Management decision outcomes for land-use systems are of high social and ecological interest but are challenging to develop, implement, and continually improve. Challenges include heterogeneous societal actors, a diversity of knowledge types, data paucity, and communication gaps, which all benefit from incorporating stakeholder insights [31,32]. Integrating scientific- and stakeholder-based knowledge streams ensures that the best available and well-validated information is used for decision making [33]. Participatory scenario development approaches are useful for assessing long-term perspectives of LULCC and for addressing the complexities and uncertainties inherent in forecasting environmental change [34–36]. Scenarios present coherent, realistic, and plausible descriptions of alternative pathways of change [37–39]. The participatory scenario development process enables researchers to guide stakeholders to collectively develop scenarios that investigate plausible futures. The process involves identifying the causes and consequences of past and present LULCC and using them to inform potential future trajectories of LULCC and their implications for livelihoods and ecosystem services. Involving diverse stakeholders in scenario development improves the quality, relevance, credibility, and legitimacy of the scenarios [40,41] and creates a sense of ownership and common understanding of the process and outputs generated. In turn, participatory approaches increase the likelihood of acceptance of the results by policy formulators and political systems [35,37]. LULCC scenarios are then combined with spatially explicit land cover models to integrate multiple and heterogeneous societal and ecological feedbacks while using stakeholder-generated knowledge in a framework that is logical, consistent, transparent, and repeatable [42,43].

Recognizing the importance of stakeholder knowledge and the challenges of exploring future outcomes of top-down LULCC policy on societies and the environment [35,44], this paper uses local stakeholder perspectives on recent and anticipated future LULCC in northern Tanzania to address three objectives. Firstly, to identify important drivers of past and present LULCC and to construct a timeline (1959 onwards) of key events that have shaped LULCC. Secondly, to use stakeholder insights of past, present and anticipated future LULCC to explore potential future (year 2030) interlinkages between LULCC, wildlife conservation, and cultural heritage conservation using scenario-based spatial model projections. Thirdly, to identify desirable and undesirable potential futures in 2030 and the implications that realizing these futures would have on meeting the SDGs and targets by 2030. The temporal scope explored the living memory of participants from 1959 up to an anticipated future in 2030. The year 2030 was selected as the time horizon for this research so that the developed scenarios of future LULCC could be aligned to the SDGs and targets for 2030, the Tanzanian national development blueprint (‘Vision 2025’), and the sustainability underpinnings of UNESCO Global Geoparks; which all promote the implementation of the 2030 SDG agenda [24].

## 2. Study area

**Fig 1:**
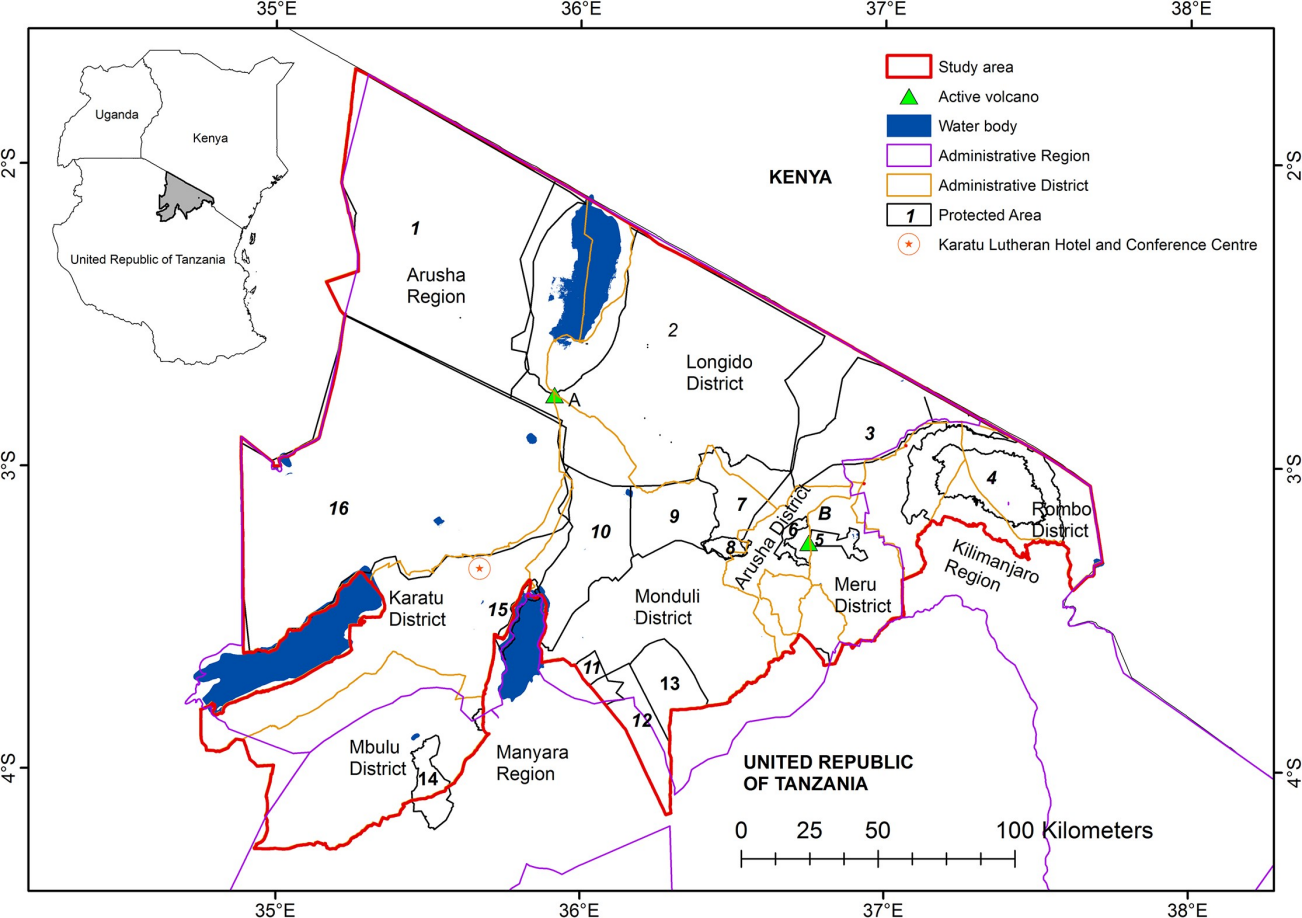
Location of the study area, workshop venue, administrative districts and protected areas. The Protected Areas are numbered as 1-Loliondo GCA, 2-Lake Natron GCA, 3-Enduimet WMA, 4-Kilimanjaro National Park, 5-Arusha National Park, 6-Meru Forest Plantation, 7-Monduli Juu Open Area, 8-Monduli Forest Reserve, 9-Burko Open Area, 10-Mto wa Mbu GCA, 11-Burunge WMA, 12-Lolkisale GCA, 13-Simanjiro GCA, 14-Nou Forest Reserve, 15-Lake Manyara National Park, 16-NCA, A-Mount Meru, B-Ol Doinyo Lengai Mountain. Protected Areas source-World Database of Protected Areas.

The physiography of northern Tanzania is largely characterized by a combination of semi-arid savanna and scrublands on the gently tilted Great Rift Valley floor and forested volcanic highlands, which are incised by river networks predominantly fed by orographic precipitation and springs [11,45]. The savannas are punctuated by barren land and large, shallow lakes in basin areas that vary in size and salinity depending on the degrees of dryness and inundation [46,47]. The climate is tropical with bimodal long (March to May) and short (October to November) rainy seasons, with notable variation across topographic zones. The highlands of Monduli District, for example, have an altitude of >2000 m above sea level and an average rainfall range of 500–900 mm yr^-1^ while the lowlands typically receive 200–600 mm yr^-1^ average rainfall [48]. The area is home to a large, ethnically, culturally, and linguistically diverse population, including people of Iraqw, Hadzabe, Warusha, Chagga, Datooga, and Maasai ethnic identities [49]. Administratively, northern Tanzania is divided into regions and subdivided into districts. The Arusha Region forms the bulk of our study area (Fig 1) and together with the Manyara and Kilimanjaro Regions, have seventeen district councils, three town councils and, a 2017 population estimate of 3,613,387 [50]. Livelihoods in northern Tanzania include smallholder agriculture, pastoralism, and agropastoralism with the overall agricultural sector providing employment to half of the population [51–53]. The highland agricultural zone on the slopes of Mount Meru largely produces coffee and horticultural crops and the lowland agricultural zone is used for maize, beans, peas, rice and livestock production. The main crops grown in the Ngorongoro highlands are maize, rice, cassava and beans. Other economic activities in northern Tanzania include mining, fishing, forestry, ecotourism, government administration, service industries, and commerce. Settlement systems include medium to large towns, rural villages, and isolated homesteads; additional land use types include designated Protected Areas, wildlife migration corridors, linear infrastructure (roads and powerlines), livestock grazing, and hunting and gathering areas [54–56]. Environmental challenges relevant to public policy and land management decision-making across the region include: climate variability [48], soil erosion [57], deforestation [58], water distribution [59], woody plant encroachment, invasive species [60,61], defaunation patterns [62], pest management [63], human-wildlife conflicts [64], population pressure on resources [65,66] and competing demands for access to land for livelihoods or cultural practices [67–70].

**Fig 2:**
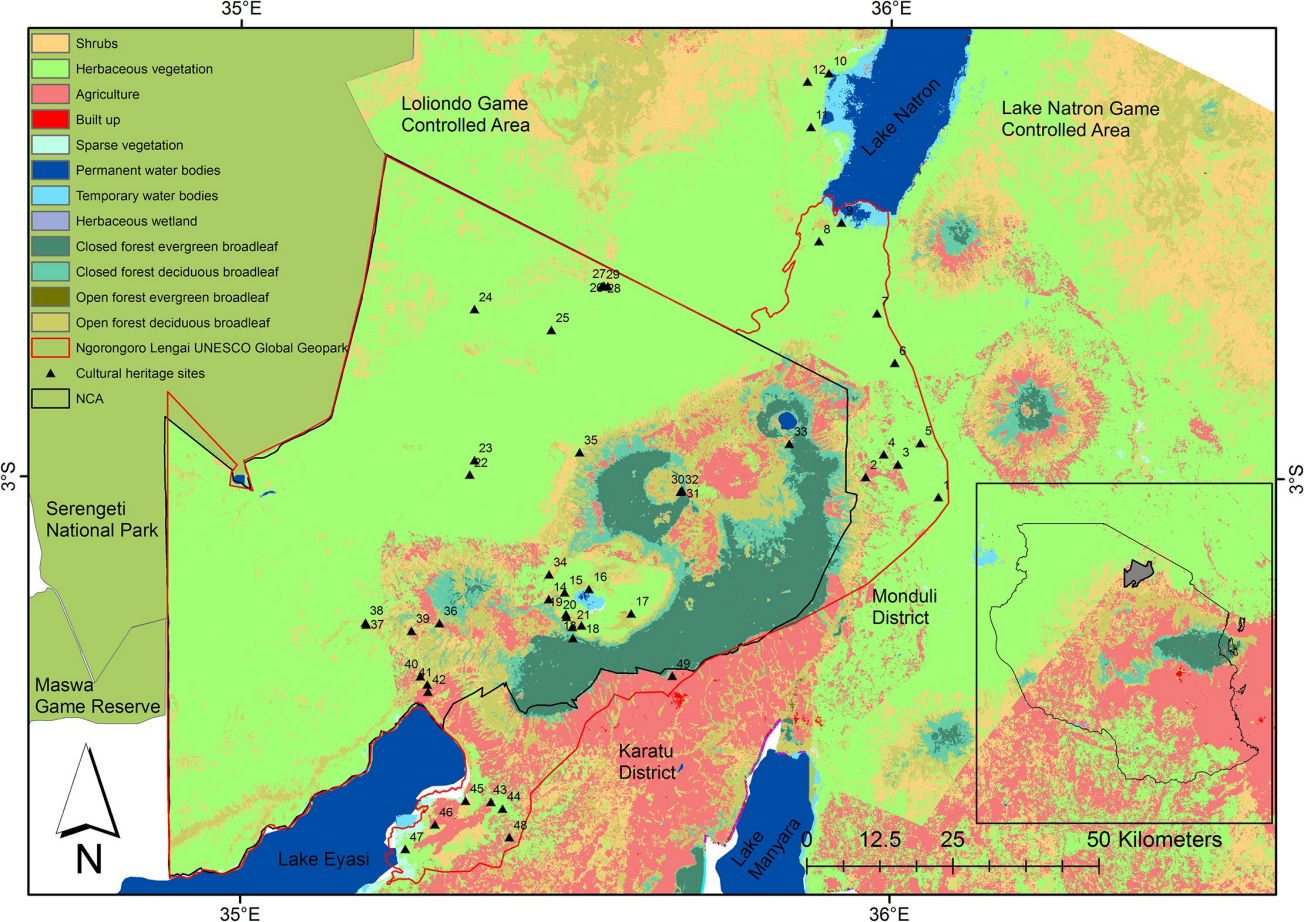
Location of officially designated natural, cultural and biocultural heritage ‘sites’ in the NCA, Ngorongoro Lengai UNESCO Global Geopark, and Lake Natron GCA, and the associated land cover in 2018. The names of the geological and cultural/biocultural heritage sites are: 1-Volcanic Pillars, 2-Engaruka Ruins, 3-Elephant Cave, 4-Empakai Engaruka, 5-Kisulisuli, 6-Shimo la Mungu, 7-Oldonyo Lengai, 8-Engaresero Museum, 9-Engaresero Footprints, 10-Engaresero HotSpring, 11-Erosion Forms, 12-Three Crown Hills, 13-Grizmek Grave, 14-Malanja Depression, 15-Seneto Spring, 16-Lake Magadi, 17-Ngoitoktok, 18-Lerai Forest, 19-Datoga Chief Grave, 20-Datoga Ritual Tree, 21-Old German House, 22-Olduvai Museum, 23-Soitoo Green-pink Quartzite Hill, 24-Nasera Rock, 25-Biotite Hill, 26-Maasai Cultural Well, 27-Olkarean Gorges, 28-Vulture Breeding Site, 29-Suspended Stone Bridge, 30-Olmoti Crater, 31-Kulangol Cultural Village, 32-Olmoti Waterfall, 33-Olcheni Lengai Empakai, 34-Seneto Cultural Boma, 35-Breathing Holes, 36-Endulen-Oreteti Tree, 37-Laetoli Museum, 38-Laetoli Footprints, 39-Traditional Beehives, 40-Gneiss Site, 41-Eyasi Geological Site, 42-Datoga Village, 43-Mangola Spring, 44-Datoga Blacksmith Site, 45-Maifola Hot Water Spring, 46-Mumba Rock, 47-Hotspring Giledabeshta, 48-Hadzabe Camp, 49-Makonde Carving. Land cover source-Copernicus Global Land Service. Protected Areas source-World Database of Protected Areas.

The total estimated terrestrial land area for our study is 42,844 km^2^ and includes all the districts in Arusha Region, Mbulu District in Manyara Region and Rombo and Siha Districts in Kilimanjaro Region (Fig 1). Ninety percent of our study area is under some form of protection and includes Forest Reserves, Game Controlled Areas (GCA), Game Reserves, National Parks, Wildlife Management Areas (WMA), and a UNESCO Global Geopark. As a Cultural and Natural World Heritage Site, the Ngorongoro Conservation Area (NCA) is northern Tanzania’s most prominent Protected Area and the nation’s most visited conservation area [15]. It exhibits multiple land uses supporting wildlife conservation, livestock keeping, settlements, internationally significant archaeological and paleontological deposits, and wildlife and cultural tourism. In the NCA, Maasai pastoralist communities settle in designated areas and keep livestock, but are not permitted to cultivate or receive land tenure. Tenure and decisions related to land, conservation, and resource use are managed by the Ngorongoro Conservation Area Authority (NCAA), which encompasses the Pastoral Council that represents the interests of resident pastoralists [55,71]. At an international level, UNESCO manages and protects cultural heritage and biodiversity of global interest within the NCA [14] and Lake Manyara National Park. The Ngorongoro Lengai UNESCO Global Geopark (henceforth referred to as ‘geopark’) was established in 2018 to sustainably manage the benefits of natural resources for local communities, and the diversity of local cultural and natural heritage resources within and around Ngorongoro [12,24,72]. It is only the second geopark to be established in Africa and encompasses the NCA and part of its surrounding area in the northeast. Places of interest within the geopark include the active Oldoinyo Lengai volcano, extinct volcanoes in the Ngorongoro Highlands and several cultural heritage sites located in forests, savannas, and agricultural areas (Fig 2). Several local institutions formalize and transmit cultural heritage, geoheritage, and traditional environmental conservation knowledge and values in the area [11,73–75]. The Loliondo Game Controlled Area (LGCA), is a neighboring multiple land use area that allows settlement, livestock keeping, smallholder agriculture (only by Maasai agro-pastoralist families), wildlife hunting, and wildlife and cultural tourism [17].

## 3. Methods

### 3.1 Ethics statement

This study was approved by the Tanzania Commission for Science and Technology (COSTECH) under permit number 2018-464-NA-2018-320 and the Tanzania Wildlife Research Institute (TAWIRI) under approval reference numbers TWRI/RS-331/VOL.III/2013/84-91.

### 3.2 Scenario development

This study employed the Kesho (Swahili word meaning “tomorrow”) participatory scenario development tool [35] to engage stakeholders and co-produce scenarios and models of land cover change and possible trajectories of change from 2018 up to 2030. Kesho is a flexible framework integrating knowledge from a diverse range of stakeholders and organizations with spatial modelling to produce qualitative, quantitative, and spatially explicit scenarios of future LULCC. A key aspect of this approach is that spatial scenario simulation follows spatial rules and predictors identified by stakeholders. This considers stakeholders’ knowledge of spatial patterns of change and provides a more explicit representation of relations between baseline socioeconomic and biophysical conditions and future LULCC. Kesho uses four steps that involve stakeholders, facilitators, and modelers: 1) stakeholders and experts collectively set scenario boundary conditions; 2) stakeholders identify future socioeconomic and environmental trajectories for each scenario; 3) experts organize, synthesize, and translate co-produced scenarios in a spatially explicit modelling platform to generate summarized scenario narratives, graphics and maps; and, 4) modelled scenarios are presented to stakeholders for feedback and validation [35,36]. Due to the Covid-19 pandemic in 2020 our study was not possible to host a stakeholder feedback and validation workshop and in lieu of the workshop, modelled scenario results were sent to a series of stakeholders for feedback followed up with a series of telephone, email, and in-person conversations.

In previous studies, the Kesho approach has been applied at a national level in mainland Tanzania to assess LULCC in 2025 under ‘business as usual’ and ‘green economy’ scenarios 35], and the potential impacts of projected LULCC on carbon stocks, diversity of terrestrial vertebrates, and water yields in 2025 [76]. On the Zanzibar Archipelago, Kesho has been integrated with other sustainability frameworks to assess interactions of food, water, energy, tourism and fishing for island communities [77]. In Kenya and Ethiopia, it has been used to assess future LULCC scenarios in agroforestry and coffee forestry [36].

### 3.3. Stakeholder selection

Stakeholders were selected from the Ngorongoro Conservation Area (NCA), and the bordering areas: Longido, Monduli, Karatu, Arusha and Mbulu Districts. Selection of the stakeholders was done collectively by the Adaptation and Resilience to Climate Change (ARCC) project researchers. The selection of stakeholders aimed to achieve equal representation of stakeholders with expertise and experience in LULCC but whose participation in academic scientific research is limited [78]. All stakeholders were adults and included farmers, pastoralists, government and nongovernmental officers, environmentalists, and researchers, but also included a road engineer, a public museum curator, a geologist, and a faith-based leader (S1 Table). Workshop invitations were communicated by telephone, followed by email and a formal letter sent through the Deputy Vice Chancellor’s Office of the Nelson Mandela African Institution of Science and Technology (NM-AIST). Prior to the meeting, stakeholders were informed of the workshop’s aims, research ethics, and personal data protection. The stakeholders then gave verbal and signed consent of their willingness to participate in the workshop. The signed consent confirmed that the participants understood the objectives of this research, had the opportunity to discuss any confidentiality issues or questions they may have had and were made aware that their anonymized responses may be used in reports and scientific publications.

### 3.4 Workshop co-production of land use land cover change scenarios

A two-day workshop (13–14 November 2018) with 20 stakeholders and researchers (six females and fourteen males) was held at the Karatu Lutheran Hotel and Conference Centre (Fig 1). Workshop activities were led and facilitated by three members of the ARCC project team (RWK, LKM and CJCM) and included presentations on Kesho’s scenario development process [35], subgroup and pairwise discussions, and feedback from subgroup and pairwise discussions to the entire group. For group activities, stakeholders were divided into three discussion groups composed of six to seven individuals of mixed professions and backgrounds to maximize each participant’s engagement with a diversity of perspectives. Participants remained in the same groups throughout the workshop.

The first day focused on stakeholder introductions, defining the scope (time interval and geographic and cultural areas of interest) (Figs 1 and 2), discussing key concepts and values, identifying and ranking past and present LULCC drivers, and developing of LULCC timelines. As the stakeholders were diverse in their professions, life experiences, ages and memories, the first activity collectively determined the respective timeframes for discussions concerning ‘the past’ and ‘the present’. Demarcations between past and present were based on key events that dramatically changed interactions among people, land use, and the environment in the study area. The aim was to achieve consensus on a common frame of reference when discussing ‘the past’ and ‘the present’. Stakeholders defined ‘the past’ as 1959–1999 and ‘the present’ as 2000–2018. The year 1959 was selected by stakeholders as the start of ‘the past’ because it was the year the Ngorongoro Conservation Area (NCA) was formed as a multiple land use area [79,80]. The year 2000 started ‘the present’ as the stakeholders considered that since 2000 there had been rapid and significant growth in tourism and infrastructure. This growth changed the tourism, socioeconomic, and governance conditions of the study area [81].

To ensure some degree of consistency and continuity in how perspectives of past and present LULCC were recorded, stakeholders were provided with a reference map that had information on the current (2018) administrative boundaries, Protected Areas, roads, rivers, and topography of the study area. The reference map was meant to provide guidance and common understanding to the stakeholders on the spatial configuration of the area under discussion. After identifying the past time frame, the stakeholders discussed and identified the main drivers of past LULCC and mapped the distribution of past land cover classes in the study area. The stakeholders then discussed and identified the main drivers of present LULCC and mapped the distribution of present land cover classes in the study area. During each exercise, the stakeholders listed the main LULCC drivers but did not explicitly identify the impact of the interactions between the LULCC drivers on the maps. The objective of mapping past and present land cover was to help the stakeholders think about how and where specific land cover classes had changed from past to present and how they are likely to change in the future.

Note that the stakeholders’ perspectives on historical LULCC were neither collected for a comparative study with actual LULCC in northern Tanzania nor were they collected to reconstruct the past and incorporate it into modelling the future. Rather, discussions of past and present LULCC aimed to elicit perspectives of historical LULCC from the living and working memories of diverse stakeholders with the intention of encouraging stakeholders to draw on their own understandings of how northern Tanzanian landscapes transition through time as they developed scenarios of future LULCC.

On the second day, workshop participants were introduced to the Kesho framework, participants co-produced scenario narratives and pathways to future LULCC, assessed the plausibility and consistency of the scenarios developed, and identified the likelihood of future LULCC and desirable and undesirable futures in 2030. The time interval for developing the future scenarios was 2018–2030. The stakeholders collectively discussed, identified, and ranked a list of possible key drivers of future LULCC, as was similarly done for past and present LULCC on the previous day. Identification and ranking of future LULCC drivers drew from discussions of past and present drivers in the study area. The drivers included climatic, environmental, socioeconomic, and governance factors. As the number of developed scenarios of future environmental change need to be feasible and manageable for discussions of future pathways of change, most studies develop up to four scenarios [42]. Thus, stakeholders collectively selected only the two most important drivers of LULCC in 2030 from all of the drivers identified. The two drivers identified were: 1) the level of economic development—defined as the degree of improvement of economic, political, infrastructural and social well-being, and 2) land health—defined as the ability of land to support the four types (provisioning, regulating, supporting, and cultural) of ecosystem services. Two extreme and opposed states for each of the two drivers were next identified. The extreme conditions identified for the level of economic development were ‘developed’ and ‘developing’ economies and for land health were ‘healthy’ and ‘degraded’ land. Finally, the extreme states were used to develop a 2 x 2 matrix [37,42]. Consequently, the four scenarios of future LULCC in 2030 co-produced by the stakeholders were: 1) developed economy with degraded land (scenario one; S1); 2) developed economy with healthy land (scenario two; S2): 3) developing economy with degraded land (scenario three; S3); and, 4) developing economy with healthy land (scenario four; S4). Under the conditions of each scenario, stakeholders developed narratives describing the overall LULCC they anticipated occurring between 2018 and 2030 and quantified how likely it was that specific LULCC would result. Thus, the four scenarios explored four alternative futures for northern Tanzania from baseline social-environmental-political conditions of northern Tanzania in 2018.

After developing the scenario narratives, stakeholders individually listed what they would most like to observe happening in the study area in 2030 and what they would least like to occur. Insights from this exercise were used to identify desirable and undesirable futures for the study area in 2030. Note that rather than simply classifying each of the four scenarios as desirable or undesirable, stakeholders instead specified the conditions that they either aspired for, or wanted to avoid living under, in 2030. The desirable and undesirable futures identified by the stakeholders, however, were not linked to the scenario narratives and thus did not classify which scenarios would be desirable and which ones would be undesirable.

### 3.5 Documenting and integrating stakeholder insight with spatial modelling

Information collected from the stakeholders included ranked lists of LULCC drivers, qualitative descriptions of future LULCC scenarios, ordered scale information on the likelihood of future LULCC, spatial information of where future LULCC is likely to occur, and a ranked list of desirable and undesirable futures. This information was captured and quantified in a spreadsheet and used to assess LULCC. Stakeholder perspectives from each discussion group on the drivers and impacts of future LULCC scenarios, scenario narratives, and spatial areas where future LULCC is likely to occur were checked, organized, integrated, and their level of influence on future LULCC was quantified in the form of ranks and percentages. Stakeholders’ narratives describing past and present LULCC drivers, and the spatial and temporal scale of this LULCC were used to identify future LULCC drivers, the likelihood of future LULCC occurring, and the areas where future land cover change is anticipated to occur. To establish the nature and area of land cover change in 2030, stakeholders were again provided with a map template which showed the extent of current (2018) land cover classes in the study area and guided to identify the type and location of land cover transformation that would occur by 2030 under each of the four scenario conditions. Identification on the map of land cover transformation projected to occur by 2030 was done after the stakeholders had formulated the narratives of change for each scenario, and so when identifying areas of land cover change, they were clear on what each scenario represented.

The modelling process did not directly involve stakeholders; rather it used stakeholder perspectives on the drivers, likelihood, and spatio-temporal scale of future LULCC for simulations for each of the land cover change scenarios. Historical land cover data were not used as input data for the spatial model but projected spatial layers of human population, crop suitability and land demand conditions from the baseline year of 2018 to the year 2030 were useful in accounting for broader scale LULCC factors that were not discussed by the stakeholders (e.g. population growth rate, and impact of projected rainfall on land cover). The data used to quantify future land demand are also based on scenarios that incorporated past trends when being developed. Preparation of the spatial layers and integration of the layers with stakeholders’ perspectives was done in ESRI ArcGIS (version 10.3), while simulation of land cover change based on future land demand for specific land classes under each of the four co-produced scenarios was done using R (version 3.5.3) [82]. Climatic, environmental, and socioeconomic spatial data associated with LULCC in northern Tanzania were obtained from multiple sources (S2 Table). Using ArcGIS, all spatial layers were clipped to the extent of the study area, then they were rasterized and their spatial resolution transformed to be uniform. From the baseline land cover map of 2018, a layer estimating the Euclidean distance of grids in the study area to cultivated land and a layer estimating the Euclidean distance of grids in the study area to the built-up areas were created. The Euclidean distance of grids in the study area to Protected Area boundaries, mining sites, and all-weather roads was estimated using spatial layers of Protected Areas, mining sites and roads (S2 Table). Using the annual human population growth rate of 2.7% for Tanzania, the human population spatial layer for the study area was projected to 2030. A projected crop suitability layer with underlying attributes of projected mean annual rainfall and temperature, pH, lithology, physiography, elevation, agroecological zone and the growing period of the crop was created. The other spatial layers were not modified.

The spatial layers were reclassified to a common scale following stakeholders’ insights on LULCC patterns and the criteria outlined by [35]. The reclassified spatial layers were then used to create composite indicator land cover change layers which identified the likelihood of different areas across the study area to change from one land cover to another by 2030. Composite indicators are formed by combining individual indicators into a single index and are useful for assessing multidimensional trends that cannot be assessed by a single indicator, as well as for signifying the direction of change across different units [83]. The composite indicator for a specific land cover class was created by linearly combining spatial layers that account for change in that land cover class while multiplying spatial layers that constrain land cover change for that land cover class. The composite indicators were then rescaled to a common scale of 1 to 5 depicting areas with low to high likelihood of land cover change for specific land cover classes.

Land demand (km^2^) in 2030 for specific land cover class in the study area was calculated based on available data on projected agriculture and livestock land demand [84], urban growth [85], and loss of forests [86] (S1 Text). For each grid cell in the baseline land cover map, a likelihood of conversion to a specific land cover/use was calculated based on a) the calculated land demand of a specific land cover class and b) stakeholders perspectives on the likelihood score of specific land cover/use demand to be met in 2030. The likelihood scores were 0 for ‘no likelihood’, 1 for ‘low likelihood’, 2 for ‘medium likelihood’, 3 for ‘high likelihood’, and 4 for ‘very high likelihood’. Then, based on specific land cover/ use demand, an equivalent number of grids was converted from a land cover class in the 2018 baseline map [87] to a new land cover starting with those with highest likelihood of change until demand was fulfilled.

The 2018 land cover map provided the baseline land cover distribution from which each of the four scenarios of future land cover change transitioned, up to the year 2030. Land cover change from the baseline 2018 land cover map to each land cover change scenario was modeled sequentially. Guided by stakeholders’ assumptions, closed forest grids could be converted to open forests, shrubland, and agricultural grids. Open forest grids could be converted to shrubland, herbaceous vegetation and agricultural grids. Shrubland grids could be converted to herbaceous vegetation, agriculture, settlements and sparse vegetation while herbaceous vegetation grids could be converted to agriculture, settlements and sparse vegetation. Densely populated areas and areas close to existing farms and roads were assumed to have a higher likelihood of agriculture transformation. Densely populated areas close to mines were assumed to have a high likelihood of being converted to sparse vegetation while built-up land cover had a high likelihood of increasing in densely populated areas with good road coverage. The model also assumed that in the study area, human induced land cover will not change inside the NCA, the Forests Reserves (Monduli and Nou), or the National Parks (Arusha, Kilimanjaro and Manyara) by 2030 because they receive the highest level of protection from the Tanzanian government due to the rich biodiversity and high plant and animal biomass. Protected areas in East Africa that are the most visited and earn the highest tourism revenues [9,15], including Tanzania’s NCA and Arusha National Park, are considered premium parks by the government and receive high priority protection from human-induced environmental change to protect biodiversity and ecosystem functions. Consequently, only non-consumptive uses of wildlife resources are permitted and hunting, settlement (besides the NCA), agriculture, and any other form of resource extraction is prohibited.

## 4. Results

This section is divided into subsections that address the objectives of the study. First, we present the drivers of past (1959–1999) and present (2000–2018) LULCC as identified by the stakeholders. Second, we present the timeline of historic events that have shaped LULCC in the study area from 1959–2018. Third, we present summarized narratives of the four future LULCC scenarios produced by the stakeholders. The main narratives for the four scenarios are presented as supporting information (S2 Text). We also present four models of land cover change in 2030 developed from stakeholder narratives of future LULCC trajectories. Finally, we discuss desirable and undesirable futures as envisioned by stakeholders capturing the climatic, environmental, socioeconomic, cultural, and political factors that would (or would not) be desirable in 2030.

### 4.1 Drivers of past and present land use land cover change

Based on knowledge acquired from living and working in LULCC in northern Tanzania, stakeholders of different ages, professions, livelihoods, ethnicities, genders and locations collectively determined historical land use land cover conditions and instances of change. Stakeholders identified the leading contributors to historical (1959–1999) LULCC patterns, in order of influence, as: variable rainfall patterns; agriculture expansion; availability of minerals (such as salt for livestock, and aggregates for construction materials); pasture quantity and quality; and volcanic eruptions (Table 1). The frequency of mentions for variable rainfall (10%) and agricultural expansion (13%), was higher than the frequency of mentions for livestock and mineral building resources (8%) and pasture quantity and quality (8%). Agricultural expansion was attributed to the nutrient rich volcanic soils in the area, and inadequate pasture was attributed to overgrazing and declining plant diversity. Besides climatic and environmental factors, governance structure was perceived as important in shaping LULCC in northern Tanzania. Since the formation of the NCA, the key post-colonial acts of legislation governing land access and natural resource management that were identified as most relevant for LULCC include the Villagization Policy (1974–1982), the Wildlife Conservation Act of 1974 (and the 1998 and 2009 revisions), the Mining Acts (1998, 2010 and 2017), and the 1999 Land Act.

**Table 1:** Identified drivers of LULCC patterns in the study area from 1959–1999 (‘the past’) and from 2000–2018 (‘the present’), and ranking in order of importance. Rank orders LULCC drivers from the driver perceived to have the greatest influence on LULCC, which is given the first number, to the driver perceived to have the lowest influence on LULCC, which is given the last number. Mentions per rank is the number of times a driver was cited by stakeholders expressed as a percentage.

Driver of past land use change	Rank (1—most important; 13- least important)	Mentions per rank (percent)	Driver of present land use change	Rank (1—most important; 13- least important)	Mentions per rank (percent)
Variable rainfall	1	10%	Human population growth	1	11%
Agricultural expansion	2	13%	Agricultural expansion	2	9%
Availability of minerals (e.g. building material and salt for livestock)	3	8%	Tourism increase	3	2%
Inadequate pasture quantity	3	8%	Urbanization and development	4	9%
Volcanic eruptions	3	5%	Deforestation, overgrazing, land degradation and soil loss	5	15%
Change of culture	6	5%	Growth in mining industries	6	5%
Pre and post-colonial acts and policies	7	36%	High education levels	7	2%
Infrastructure development	8	10%	Poor governance and policy implementation	8	7%
Social-economic development	9	5%	Government policies	9	13%
		100%	Climate change impacts	10	18%
			Culture disappearance	11	4%
			Volcanic eruptions and earthquakes	12	4%
			Biodiversity loss	13	2%
					100%

Stakeholders identified and ranked the most important drivers of present (2000–2018) LULCC, in order of influence, as human population growth, agricultural expansion, tourism increase, and urbanization (Table 1). Cultural loss was also mentioned, and included loss of cultural laws and fragmentation of cultural heritage sites. Land degradation was an umbrella concept mentioned by stakeholders as encompassing overgrazing, deforestation, and soil erosion. Other factors, such as rainfall variability and volcanic activity, that were ranked as very important drivers of past LULCC were not ranked as being very important to present LULCC. Overall, LULCC drivers such as livestock, the extraction of mineral resources used in building, government policies, volcanic eruptions, and cultural change were perceived to account for both past and present change.

### 4.2. Timeline of key events influencing LULCC

**Fig 3:**
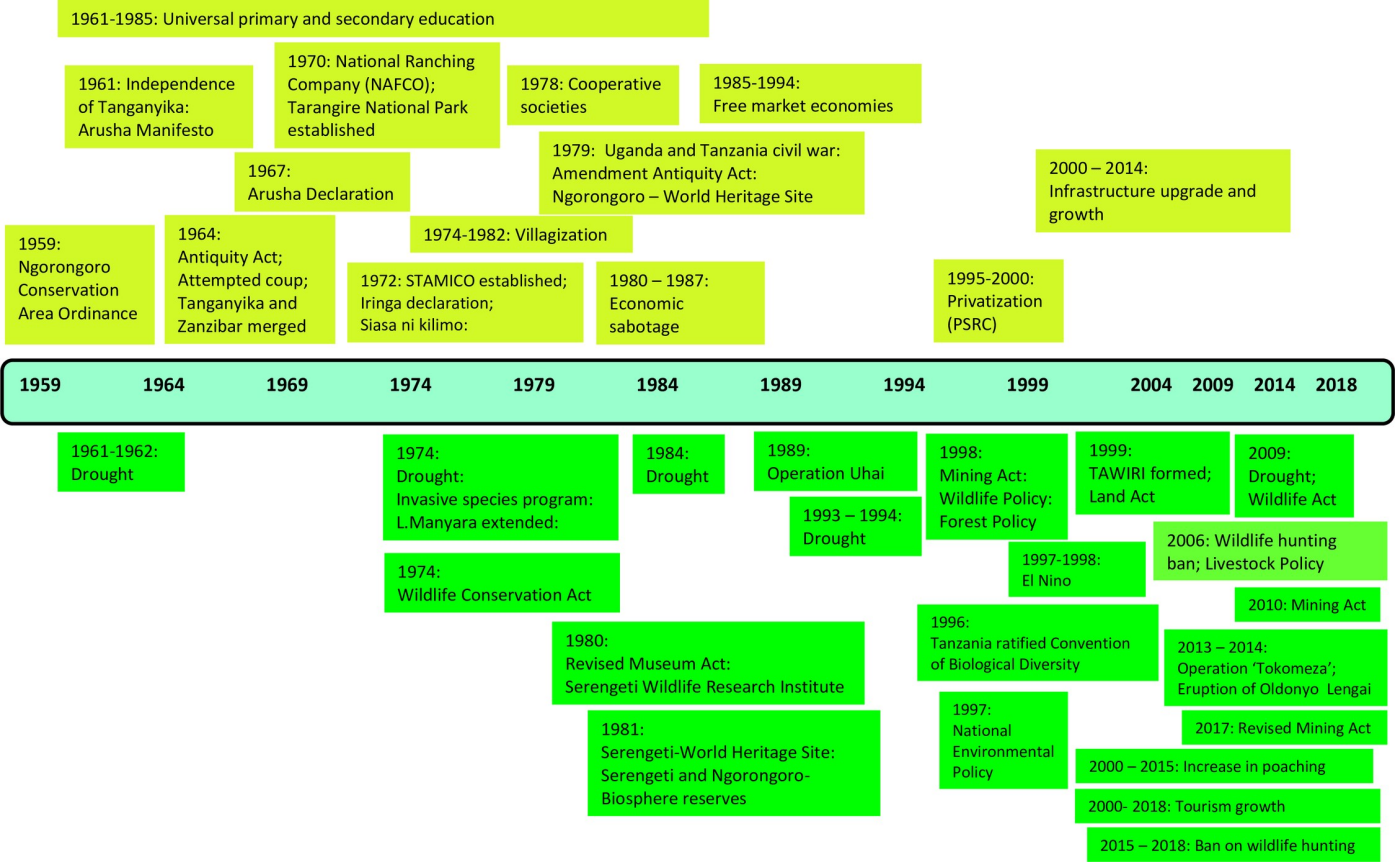
Timeline of events identified by stakeholders as key in shaping LULCC in the study area from 1959 to 2018. Color shades represent events with socioeconomic (light green) or environmental (dark green) impacts.

The stakeholders used their lived recollections to generate timelines of significant events that shaped LULCC in the study area from 1959–2018 (Fig 3). Unless otherwise cited, all events in this section were identified by the stakeholders. The start date chosen was 1959, when the NCA was formed and designated a multiple land use area under the Ngorongoro Conservation Ordinance. Formation of the NCA resulted in the relocation of pastoralists from Serengeti National Park, Moru, and Sironet Springs to the NCA [17,58,79]. Overall, key events that shaped LULCC (agricultural expansion, human settlements, establishment of wildlife and cultural-heritage conservation areas, infrastructure expansion etc.) from 1959–2018 were linked to climatic, volcanic, wildlife conservation, governance, and economic factors. Immediately following independence, the national government established universal healthcare and primary and secondary education, and the associated benefits were thought to be important drivers of LULCC up to 1985. Beginning in the 1960s, legislation was perceived to have strongly governed activities influencing LULCC patterns. The Arusha Declaration of 1967 and villagization schemes that began in the 1970s were prominent in driving human settlement and agricultural trends in the study area. The Mining Acts of 1998, 2010, and 2017, Antiquity Act of 1964 (revised in 1979), National Museum Act of 1963 and 1980, Wildlife Conservation Act of 1974 and 2009, and Wildlife Policy of 1998, together with the establishment of Protected Areas and invasive species management programs in the 1960s and 1970s, were identified as significant in governing the management of mining, cultural preservation, and wildlife and forest conservation areas. Wildlife conservation legislation was also viewed as the cause of land use conflicts between pastoralists and agriculturalists, and the ratification of the Convention on Biological Diversity by Tanzania in 1996 was viewed as key in shaping the conservation and sustainable use of biological resources in the study area. Interestingly, the legacies of previous game ordinance acts and limitations on hunting beginning half a century earlier [88] were not a major point of discussion. During the 1980s and 1990s the formation of environmental research organizations, such as the Serengeti Wildlife Research Institute (1980, and renamed in 1999 to Tanzania Wildlife Research Institute), and the revision and implementation of existing and new acts and policies were reported as key in shaping LULCC. Generally, from the 2000s, tourism and infrastructure development were associated with initiating growth in wildlife conservation areas, wildlife tourism infrastructure and markets for agricultural products.

Since 2000, resurgent poaching levels and regrowth in tourism and new infrastructure were considered key drivers of LULCC in the study area (Fig 3). Stakeholders associated infrastructure growth with the National Investment Policy of Tanzania that involved the private sector and foreign aid in developing key infrastructure. Additionally, the growth in infrastructure was linked with policies that promoted infrastructure development, such as the National Transport Policy (2003) and the Construction Industry Policy (2003). Roads, such as that joining Loliondo and Mto wa Mbu, and joining the Arusha and Mara Regions, which provide the main gateway to the NCA and Serengeti National Park, were reported to be paved during ‘the present’ interval. Manyara Region was also established as an administrative entity during this time after being separated from Arusha Region in 2002. The stakeholders associated tourism growth with a surge in visitors, higher tourism revenue for Protected Areas in northern Tanzania, and an increased number of accommodation facilities and roads inside Karatu town and in the Protected Areas in the region. Tourism growth was also associated with increased ecotourism initiatives in the area and the use of community land, in the form of Wildlife Management Areas (WMA), for wildlife conservation and tourism. High poaching levels between 2000 and 2015 that led to the enactment of a ban on local hunting in 2006, and the establishment of ‘Operation Tokomeza’ in 2013–2014 as an effort to end the poaching of large mammals in Tanzania, were reported to drive LULCC around Protected Areas resulting in human encroachment and land-use conflicts between conservationists and communities. Droughts and volcanic eruptions were largely perceived to drive livestock grazing and farming patterns and those that were remembered to have occurred between 1959 and 2018 were added to the timeline; although several other volcanic eruptions have been confirmed during this time [89].

### 4.3 Scenarios of future land cover change

**Fig 4:**
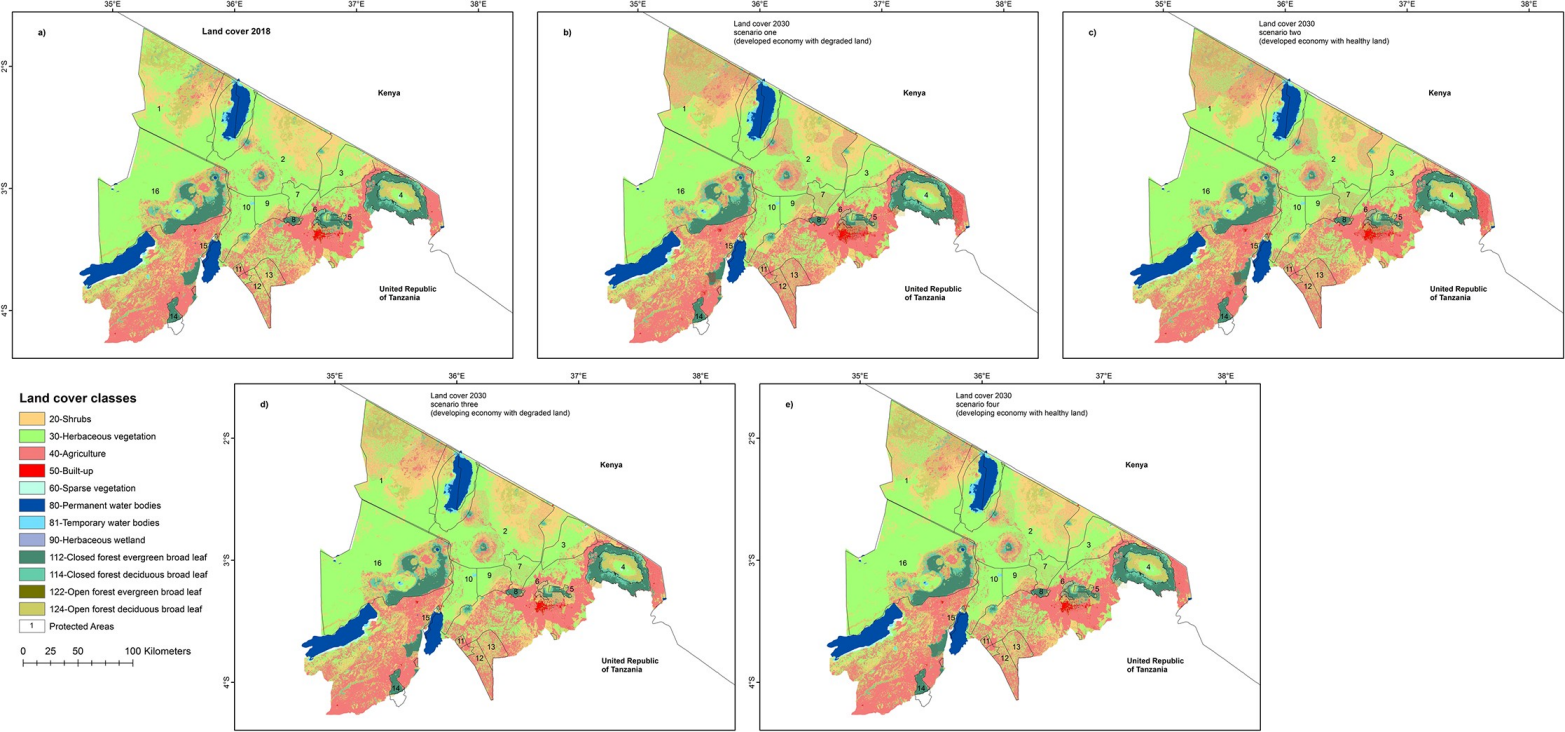
**(a)** Land cover categories in northern Tanzania in 2018 **(b)** and anticipated future land cover change in 2030 as envisaged under scenario one: developed economy with degraded land, (**c)** scenario two: developed economy with healthy land, **(d)** scenario three: developing economy with degraded land, and **(e)** scenario four: developing economy with healthy land. The Protected Areas are numbered as 1-Loliondo GCA, 2-Lake Natron GCA, 3-Enduimet WMA, 4-Kilimanjaro National Park, 5-Arusha National Park, 6-Meru Forest Plantation, 7-Monduli Juu Open Area, 8-Monduli Forest Reserve, 9-Burko Open Area, 10-Mto wa Mbu GCA, 11-Burunge WMA, 12-Lolkisale GCA, 13-Simanjiro GCA, 14-Nou Forest Reserve, 15-Lake Manyara National Park, 16-Ngorongoro Conservation Area (NCA). Land cover source-Copernicus Global Land Service. Protected Areas source-World Database of Protected Areas.

Under the first scenario which envisions northern Tanzania will be further developed than it is now, but have poor land health in 2030, ‘top-down’ land-use plans that will disregard participatory approaches from land use stakeholders will be used. There will also be political inefficiency or lack of political will to implement land use plans leading to unplanned land uses, and ineffective enforcement of regulations governing community Protected Areas, mainly the GCAs and WMAs. Urbanization will be unplanned and will increase near existing towns, settlements, and densely populated agricultural areas. In conjunction with urbanization, infrastructural growth will improve access in the area and will promote agriculture expansion near existing farms in wet areas, settlements, and forests. In comparison to the land cover in 2018 (Fig 4A), major agricultural expansion in 2030 is projected to occur in the northwestern part of Loliondo GCA, the northern parts of Lolkisale and Simanjiro GCA, Longido GCA, Monduli and Burko Open Areas, and parts of Mbulu and Arusha Rural Districts (Fig 4B). Across the Protected Areas, agricultural expansion of 39km^2^ at Enduimet WMA and 19 km^2^ at Burunge WMA will be lower than at the GCAs (apart from Mto wa Mbu) and the Open Areas (S3 Table). Besides agricultural expansion, shrublands will expand by 44km^2^ outside Arusha and Kilimanjaro National Parks, and at the Mto wa Mbu and Loliondo GCAs. Expansion of the built-up area will be in Mbulu and Rombo Districts, and near Arusha town. Increase in agricultural, shrubland, and built-up areas will lead to a loss of 901 km^2^ of herbaceous layer and 1247 km^2^ of open and closed forests (Table 2). Sparse vegetation and bare ground under this scenario will increase by 6 km^2^. As most known cultural heritage sites are located inside the NCA, the geopark, or in remote and dry grasslands and woodlands, most will not undergo land transformation by 2030. However, agricultural and urban expansion will pose threats to cultural heritage sites that are currently undocumented. Moreover, land cover around the Empakaai Engaruka cultural heritage site (site 4: Fig 2) will be transformed from grassland to cropland in all the scenarios as the site is located along a river making the area suitable for irrigated wheat, maize, millet, and legumes, likely resulting in damage to the surviving standing remains and buried deposits. In all the scenarios, the area around the Datoga Ritual Tree (site 20: Fig 2) will be transformed from a forest to a shrubland, posing threats to the cultural value of the site.

**Table 2:** Area for land cover categories (km^2^) at the 2018 baseline map, and for the four land cover change scenarios in the year 2030.

Land cover code and description	Area (km^2^) in 2018	Area (km^2^) in 2030			
		Scenario one	Scenario two	Scenario three	Scenario four
20 –Shrubland	6634.01	6677.40	6433.11	7361.51	6705.13
30—Herbaceous vegetation	17567.60	16666.26	16758.96	17112.39	17151.93
40 –Agriculture	9570.50	11484.92	11311.57	10577.60	10495.83
50—Built-up	130.51	339.97	290.68	168.83	169.70
60—Sparse vegetation	110.33	115.90	110.33	115.87	112.82
112—Closed forest evergreen broad leaf	2208.62	2093.35	2138.15	2068.94	2154.70
114—Closed forest deciduous broad leaf	1220.46	1059.04	1111.29	1039.04	1141.36
122—Open forest evergreen broad leaf	6.61	4.80	5.67	4.19	5.82
124—Open forest deciduous broad leaf	4860.29	3892.02	4162.10	3895.49	4383.21

The second scenario envisions a developed society with healthy land. This scenario prioritizes effective governance structures, growing market access, better infrastructure, and heightened environmental awareness in improving the socioeconomic status of societies and the environmental integrity of landscapes in northern Tanzania. Agricultural expansion will also be regulated to avoid encroachment in sub-arable conservation rangelands. Urbanization will be planned and regulated by the district councils, and will occur in the densely populated locales near existing urban areas and settlements. This scenario will aim to balance economic development with environmental integrity; thus agricultural lands will expand in the same areas as those in scenario one but the expansion will be less extensive. Agriculture will expand in wet and fertile areas suitable for wheat, maize, sorghum, sunflower, potato, and legumes farming. The built-up area will grow by 161 km^2^ near Arusha town, and Mbulu and Rombo Districts, and there will be a loss of 201 km^2^ of shrubland, 809 km^2^ of herbaceous layer, and 877 km^2^ of forest cover (Fig 4C and Table 2). In the GCAs, WMAs, and protected Open Areas, less area covered by shrublands, herbaceous vegetation, or forests will be lost compared to scenario one because of lower rates of agricultural expansion (S3 Table). For cultural heritage sites, as in scenario one, only the Engaruka Empakaai site will be transformed from grassland to an agricultural area by 2030. With better integration with the planning system and anticipated enhanced provision for environmental and archaeological impact assessments, the threats posed to both known and undocumented cultural heritage will be easier to control and mitigate, helping to ensure future sustainability of these assets.

The third scenario portrays the study area as having a developing economy and poor land health. In this scenario, the formation of policies and regulations for natural resource and cultural heritage management will disregard participatory approaches, leading to inefficient land use policies. Good natural resource management policies will either not be implemented or be poorly implemented. This will lead to overgrazing, deforestation, and the expansion of smallholder agriculture in wet, fertile, densely populated areas with relatively good road infrastructure including: the unprotected forests in Loliondo GCA; Burko and Monduli Juu Open Areas; near Arusha and Kilimanjaro National Parks; and the shrublands in Monduli and Loliondo Districts. The forested and herbaceous vegetation zones are projected to decline by 1288 km^2^ and 416 km^2^ respectively, while shrublands, agricultural lands, and sparsely vegetated areas will expand by 727 km^2^, 1007 km^2^, and 6 km^2^ respectively (Fig 4D and Table 2). Shrubland expansion under this scenario will be a consequence of climate change, high deforestation rates, and overgrazing by livestock. Future land cover change trends in this scenario also project a decline in herbaceous vegetation and forest cover in the Protected Areas (S3 Table). However, the decline will be lower than in scenarios one and two because of lower agricultural land conversion. In the absence of effective land use planning and a functioning regulatory framework, documented and undocumented cultural heritage will be equally at risk from multiple anthropogenic threats.

In the final scenario, northern Tanzania will have a developing economy with healthy land. In this scenario participatory approaches for developing land use plans, which are foreseen as providing great guidelines for land use planning, will be implemented. There will be continued engagement in environmentalism, and commitment to sustainable wildlife and cultural conservation through sharing income from wildlife and cultural tourism with local communities. Existing mechanisms for protecting cultural sites will continue (including traditional custodianship of some), but without additional investment or enhancement of their potential contributions to the local economy, education, and/or societal well-being. Infrastructure development will only be for key roads and projects needed by the communities, and will occur close to existing urban centers in the Kilimanjaro and Arusha areas. Forest cover will decline by 611 km^2^, and shrublands and agriculture will expand by 71 km^2^ and 925 km^2^ in the southern parts of Arusha District, Loliondo and Longido GCAs, and the northern parts of Lolkisale and Simanjiro GCAs (Fig 4E and Table 2). The urbanized area will expand by 40 km^2^. Forest and herbaceous loss in the Protected Areas under this scenario are lower than in scenario three (S3 Table).

Feedback and validation of the modelled scenario outputs from a subset of stakeholders, comprising ecologists, a geologist, roads engineer and a tourism government officer, largely confirmed that the modelled outputs reflected the original workshop participants’ views.

### 4.4 Desirable and undesirable futures in 2030

**Fig 5:**
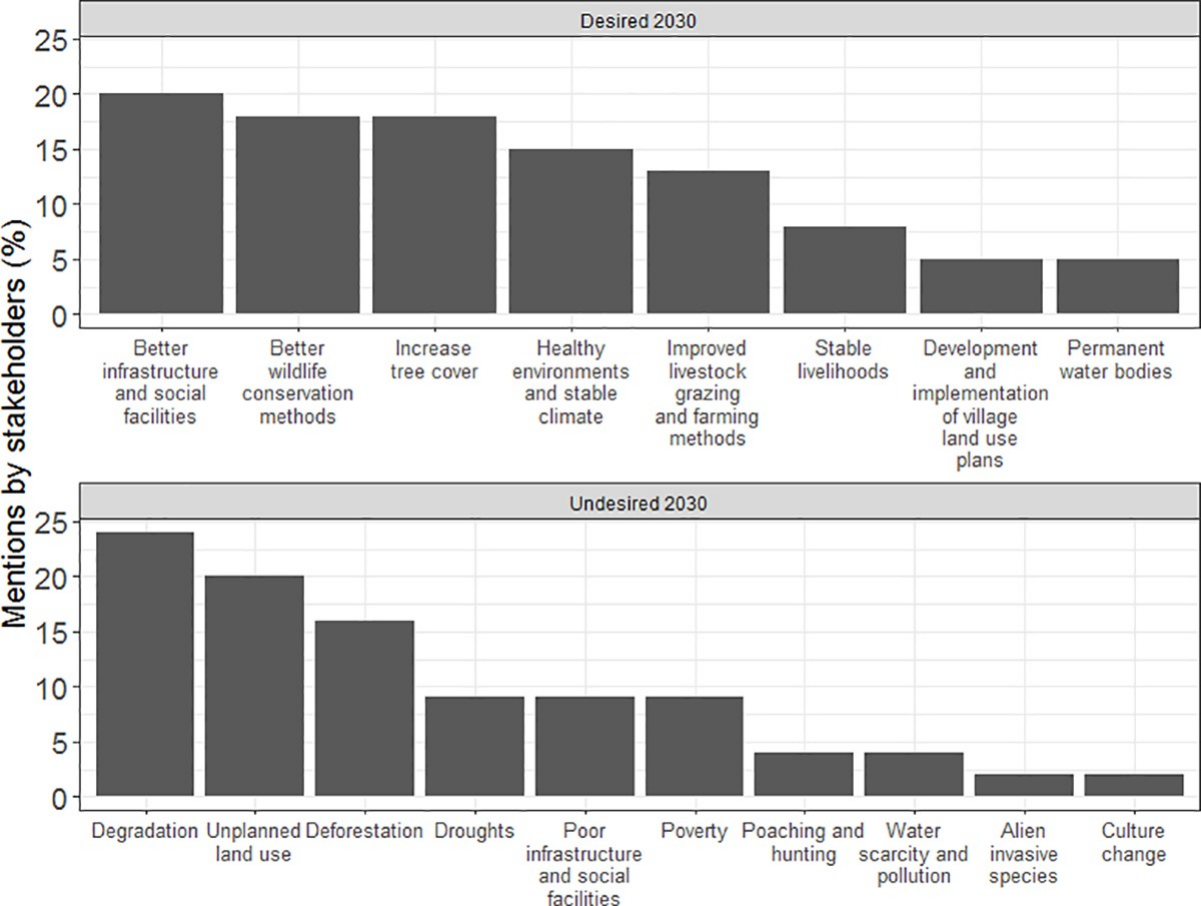
Stakeholders’ perceptions of desirable and undesirable aspects of socioeconomic development and land use and land cover changes in the future (up to 2030).

In accordance with the scenarios developed by the stakeholders and the identified major drivers of future LULCC from 2019 to 2030 in our study area, the future desired by stakeholders in 2030 was one characterized by good infrastructure, high environmental integrity, and stable livelihoods. In terms of infrastructure, by 2030, 20% of stakeholders would like to have the existing road networks improved and maintained to a high standard, as well as see the development of good water infrastructure, improved access to markets, provision of social facilities (such as schools, hospitals and community centers), and an optimization of renewable energy resources (Fig 5). Expounding on the concept of land health from the co-produced scenarios: 18% of the stakeholders would like to see an increase in tree cover in 2030 and specifically the restoration of the highland forests of Ngorongoro; 18% would like to see the improvement of wildlife conservation practices and the extension of tourism benefits to local communities; and 13% would like to see controlled livestock grazing in the study area, including inside the NCA, and the prevention of irrigated agriculture along riverine areas. With regards to improved farming techniques, the stakeholders would also like to see the increased use of contour farming to avoid soil erosion, farming techniques that ensure food security, and good governance structures that prohibit unsustainable farming.

What the stakeholders did not want to occur in 2030 was generally, land degradation, low levels of socioeconomic development, and increases in poverty. In agreement with the scenarios depicted by degraded land, the stakeholders associated degraded land with overgrazing, deforestation, uncontrolled mining, charcoal burning, soil erosion, invasive species, agriculture along water bodies, and overexploitation of forest resources. Twenty four percent of the stakeholders wished there would be no land degradation in 2030, 16% did not want deforestation to continue, and 2% wished alien invasive species would be eradicated by 2030 (Fig 5). Nine percent of the stakeholders did not want to have poor infrastructure and social facilities, 9% wished there would be no poverty, and 2% were keen to preserve their cultural identity in 2030. Although 9% of the stakeholders wished that droughts would not occur in 2030, they expected deforestation, land degradation, and soil erosion would be halted and consequently, the impact of droughts on food and livestock production as well as wildlife conservation would be minimized.

## 5 Discussion

Identifying and addressing research gaps that promote sustainable economic and sociocultural development requires the collaboration of stakeholders from all sectors of society. Through our stakeholder driven approach of developing scenarios of future LULCC, we show the utility of landscape-level approaches in assessing and forecasting environmental change and potential pathways to sustainable development. We discuss key LULCC drivers identified by stakeholders in our study area (section 5.1), and the implications of scenarios of future LULCC on wildlife conservation, cultural heritage, and sustainable development (sections 5.2–5.4).

### 5.1 Key drivers of past, present and future LULCC in northern Tanzania

From stakeholders perspectives, past (1959–1999) LULCC in northern Tanzania was largely driven by rainfall patterns, agricultural expansion, and the availability of natural resources such as pasture and water, while present (2000–2018) LULCC was largely driven by socioeconomic changes in human populations, agricultural expansion, tourism, and urbanization. LULCC in the next ten years will mainly be driven by land health, the state of natural and economic capital, and political will in planning and implementing land use policies. Documented LULCC drivers in northern Tanzania between 1959 and 1999 include a rinderpest epidemic, droughts, agricultural expansion and the Arusha Declaration of 1967 [17,90,91]. Documented LULCC drivers between 2000 and 2008 include human population growth, agricultural expansion, tourism growth, land degradation and government policies [9,75,92–94]. We discuss key LULCC drivers identified by stakeholders and their connection to the future land cover change scenarios for northern Tanzania.

Variability in rainfall amount and seasonality is a key determinant of LULCC in northern Tanzania as it drives livestock grazing, agropastoralism and agricultural livelihood decisions. Over the past and present time frames covered (1959–2018), 13 documented severe droughts (Fig 3) [48] and four extremely wet years [91] heavily impacted pastoralists and farmers [48]. The 1961–1962 drought caused widespread livestock losses to pastoralists who had previously forfeited dry season grazing lands and permanent wetlands to wildlife conservation and convinced northern Tanzanian pastoralists to farm [90]. The 2009 drought was associated with decreased woody savanna [94] and prompted extensive pastoral movements from southern Kenya into northern Tanzania as livestock herders sought water and pasture [95]. Future climate projections for northern Tanzania predict disrupted rainfall seasons, higher frequency and severity of drought and flooding events, and a reduction of rangeland vegetation biomass [96], implying climate will be important in shaping LULCC. The scenarios forecast major land cover changes in 2030 will be concentrated in wet areas without high priority protection such as the northwestern parts of the Loliondo GCA, southwestern Monduli District, and elevated areas near Mount Meru and Kilimanjaro in Arusha and Rombo Districts. The type and effectiveness of protection will therefore be a key determinant of the extent of LULCC in wet areas of northern Tanzania.

The influence of government legislation on LULCC in northern Tanzania has largely been through policies that favor sedentary agricultural communities in the provisioning of infrastructure and markets [40], while associating pastoralism with overstocking and rangeland degradation [6,97]. The Villagization Policy of 1974 aimed at organizing rural populations into designated villages, discouraged mobile pastoralism and instead promoted subsistence agriculture and sedentarization [90,91,98]. The Wildlife Conservation Act of 1974 was another policy of significance to LULCC, as it removed access for herders to large swathes of grazing land in Ngorongoro, Serengeti and Tarangire National Park by reserving these for wildlife conservation [90]. Under this Act, 605 km^2^ of land previously managed by pastoralists were designated Game Controlled Areas, with all land use decisions now being the purview of the Ministry of Natural Resources and Tourism [58]. In the 1990s, land law reforms came to be increasingly driven by market economy principles, with a large degree of oversight from international donors. Conflicts over land use resulting from land law reforms in the 1990s were remembered by workshop participants as being acute. The Land Policy seemed to associate pastoralism with encroachment on agricultural lands and contestation over natural resource use. Tanzania has an estimated 30.5 million cattle, 18.8 million goats and 5.3 million sheep [99]; yet, pastoralists continue to be marginalized from decisions relating to the use and management of land, despite their livelihoods depending directly on natural resources and mobility within the landscape [95]. Stakeholders envisioned the marginalization of pastoralists will continue up to 2030 and combined with changing climates, socioeconomic factors, and fragmentation of grazing areas, the extent of livestock grazing in 2030 under all four scenarios will continue to decline. However, livestock grazing intensities will be higher in scenarios three and four, where average rates of agricultural expansion (9%) and loss of herbaceous vegetation (2.5%) will be lower than those under scenarios one and two (10% and 5% respectively). Sparse vegetation under scenario three and four will, however, be higher than scenario two (Table 2) which will be characterized by higher environmental integrity. Institutions and governance structures will nonetheless continue shaping rangeland LULCC and there will be a need for coordinating natural resource management policies to accommodate the interests and rights of diverse land users, and for identifying sustainable future pathways.

In the NCA, historical legislation was rooted in colonial policies and reflected in early fortress conservation approaches [88,100] that evicted pastoralists from Ngorongoro Crater in 1954, and the Serengeti National Park, which is north of the NCA, in 1958 [17,90,101]. Following their eviction, and the subsequent inaccessibility of their best livestock grazing land and water resources [102], many pastoralists moved into the NCA where numbers have since increased from an estimated 6,000 in 1959 to over 65,000 in 2014 [103]. As the NCA attracts visits from 50% of all international tourists to Tanzania [9], is connected to the Serengeti National Park and the Loliondo Conservation Area, and recently received Global Geopark status the scenarios assume there will be no human induced LULCC across the NCA by 2030 due to the high level of protection and enforcement it will continue receiving from the national government. This implies that LULCC in the NCA and the geopark in 2030 will be shaped by government policies, management interventions, and LULCC outside the NCA.

Agriculture is a major component of the Tanzanian economy [104], accounting for 28% of the Gross Domestic Product (GDP), and supporting 80% of livelihoods [105]. Currently 30% of Tanzania’s land is cultivated, mainly through rain-fed, subsistence agriculture [106] although 32% is suitable for irrigation development [107]. Across northern Tanzania, most communities were engaged in some form of farming for centuries with agriculture expanding since the 1930s [90] and significantly faster in wet areas compared to drier areas [91]. Between 1984 and 2000, agriculture expanded by 520 km^2^ and 362 km^2^ in Monduli and Simanjiro Districts [91]. Additionally, agricultural production in Simanjiro District increased by 34% between 2002 and 2003 [102]. Across the Serengeti ecosystem, the neighboring Protected Areas and the surrounding 30 km buffer area of communal land, agriculture expanded by 1408 km^2^, and grasslands by 3629 km^2^, while woodlands declined by 6766 km^2^ between 1975 and 2015 [93]. All four scenarios project a growth in agriculture by 2030. As most (90%) of the study area is protected, agricultural expansion will be linked to pro-sedentarization policies, and economic pressures that encourage livelihood diversification to protect land from alienation for wildlife conservation or from large scale agricultural operations [90,102]. For instance, stakeholders identified the Iringa Declaration of *Siasa ni Kilimo* (Politics is Agriculture) in 1972 promoted sedentarization policies that created awareness and expansion of agriculture in northern Tanzania. Agriculture is forecasted to expand in Rombo (near Kilimanjaro), parts of the Lolkisale, Simanjiro, Lake Natron and Loliondo GCAs, and in Arusha Rural District. Large-scale farming and permanent subsistence agriculture is already prevalent in the northern part of Lolkisale GCA and the Simanjiro Plains, where it has been associated with the insularization and decline of wildlife in Tarangire National Park [108]. Open shrublands and woody savannas in community areas surrounding Tarangire National Park, and including Lolkisale GCA and Mto wa Mbu GCA are documented to have reduced by 50 km^2^ and 100 km^2^ between 1988 and 2009 [94]. In Loliondo, livestock production has been prevalent though smallholder agriculture is becoming important for many households [97]. In the NCA, there was a ban on agriculture from 1975–1992 (though encroachment into forests for small plot cultivation continued to occur) that was reinstated in 2009 [9,17]. Regulatory and legislative uncertainty leads to livelihood insecurity and reliance on other strategies such as ecotourism. Given the annual rate of population increase (2.7%) for Tanzania, if the efficiency of legislation protecting conservation areas in the NCA is reduced, our scenarios show that human population and agriculture would expand in the wet and fertile northern highlands of Ngorongoro by 2030, an area currently reserved for wildlife conservation.

Tourism in Tanzania is almost entirely wildlife based and is the second largest revenue earner after agriculture [102]. Tourism contributed 9.9% of Tanzania’s GDP in 2013 [15] and 9% in 2017 and, until the Covid-19 pandemic in 2020, revenues were projected to increase in the coming years [109]. Stakeholders identified that exponential tourism growth between 2000 and 2018 led to higher tourism revenues, an upgrade in the road network, and the approval by the Tanzanian government for the formation of Wildlife Management Areas (WMAs) on community land. Currently, Tanzania has 38 WMAs which are estimated to cover 7% of the country’s land surface [110]. The Serengeti-Ngorongoro ecosystem receives 50% of all international tourists travelling into Tanzania [111] and brings in at least $100 million USD in tourism revenues [105]. In 2016, the NCAA received approximately $70 million USD in tourism revenue from entrance fees [109]. In Loliondo, the Tanzanian government earns tourism and hunting revenue from village land by granting hunting concessions to investors. Several villages with ‘Village Certificates of Land’ have liaised with investors to establish tourism ventures on their land that benefit both investors and residents [97,103]. Tourism ventures on village lands generate annual revenues of up to $50,000 USD [112]. With the scenarios indicating there will be forest loss in Loliondo in 2030, deforestation and fragmentation of forests would reduce the connectivity between wildlife habitats. Consequently, the loss of forest in Loliondo might negatively affect its tourism potential given its proximity to Serengeti, diverse wildlife, and geological features such as kopjes [108]. Addressing the sustainability of future landscapes would therefore require integrated policies that address LULCC, biodiversity conservation, land management, cultural heritage protection, and livelihood support.

Infrastructure growth and development was identified as key in opening up access to markets and remote areas in northern Tanzania from 2000 to 2014. Market and road access expanded agricultural and wildlife conservation land uses. In the first decade following Tanganyika’s (now mainland Tanzania’s) independence from the British colonial government in 1961, the nation was widely lacking basic infrastructure and well-established administrative and private institutions [107]. The newly independent government prioritized the formation of necessary government institutions, provision of formal education, and national social and economic development plans, including a number of environmental protection policies (Fig 2) that influenced land use patterns in northern Tanzania. The Arusha Declaration of 1967 was highly discussed among participants because of the social policies intended to promote mass nationalization, social-economic liberation, peace and stability [104]. In the 1960s and 1970s, Tanzania was a young, independent nation that relied on borrowed funds to develop its economy and by the 1980s, when it was undergoing its transformation from a socialist to more neoliberal state [102], it faced acute shortages of foreign exchange, and huge budget deficits [107]. Stakeholders at the workshop described 1980–1987 as being tantamount to economic sabotage with limited land use transformations.

### 5.2 Interactions between future LULCC, wildlife and cultural conservation

Common challenges that face developing countries, such as widespread poverty, rapid population growth, food insecurity, and political instability, threaten the management of protected areas and biodiversity conservation in Tanzania [113]. Areas rich in natural resources may be prioritized for environmental conservation, which can often modify tradeoffs over food security, and access to water and energy resources for local peoples [114]. From the 2030 scenarios, Protected Areas on the slopes of Kilimanjaro, the highland forests in NCA, and part of the Meru highlands in the Arusha National Park, were the most suitable for agricultural conversion given their topographic, climatic, and edaphic factors, as well as access to perceived markets. However, as these areas currently receive the highest protection by the Tanzanian government, in all our scenarios, stakeholders perceived it will be unlikely for them to be converted for agricultural land use by 2030 under existing policy. In addition, >20 formally recognized cultural heritage sites are located within the geopark (Fig 2)—where livestock grazing and agriculture are currently prohibited. Other documented cultural heritage sites outside the geopark boundaries are in the dry grasslands of Monduli District, Longido District, and around Lake Natron. Our scenarios found these grasslands too dry and sparsely populated with poor infrastructure to support significant urban and agricultural growth by 2030, meaning the conservation of cultural heritage sites outside the geopark will more likely be determined by cultural, management, socioeconomic and governance changes.

Overall, the principles for managing Protected Areas for effective biodiversity conservation should consider the needs of residents adjacent to Protected Areas, the integration of socioeconomic development with biodiversity conservation, the forging of links between conservation and other sectors of the economy (including heritage industries), and the development of positive relationships with local communities [115–119]. The scenarios indicate that agricultural expansion will occur in areas of the GCAs in Loliondo, Lolkisale, and Simanjiro that are currently used for wildlife conservation. This means, an integrated approach incorporating local to global governance structures is necessary for managing the landscape more transparently and with evidence-based approaches [120]. The formation of the geopark, which is managed by the NCAA and UNESCO, is a step toward addressing these principles as its existence promotes the sustainable development of communities as well as conserving natural resources across the entire landscape. Community designed and managed approaches to cultural heritage protection, especially the formulation of biocultural protocols, could have a significant impact on blending these dual goals [20,121].

### 5.3 Link between existing land use land cover with desirable and undesirable futures in 2030

Healthy environments characterized by improved wildlife conservation methods, less challenging climate change impacts, high areal tree cover, and reduced risks of land degradation and invasive species, were especially desired by the workshop participants for 2030. In part, this might be because grass biomass and quality, especially in Monduli District, has declined and soil erosion on barren land has worsened, as evidenced by localized erosion scars and gully erosion over the last two decades [48,75]. The quality, quantity, and spatial distribution of natural pastures in the study area have been degraded by anthropogenic pressures in part in response to increased climatic variability in the area. No wonder stakeholders wished there were no droughts, water scarcity, or pollution in 2030. Calls to address land use, resource availability, and climate variability in eastern Africa [68,122], and in the NCA in particular [123], have been made. Recently, invasive species management initiatives have been developed and integrated into the management policy of the NCA. Effective land management, however, requires balancing resource availability and human activities through institutions that monitor resource distribution and use across diverse communities, and at different spatio-temporal levels [124,125].

Conservation of cultural heritage sites and cultural practices was a desirable attribute for the future; in part because pastoralists and hunter-gatherer communities are increasingly perceived in the study area as having prevented land degradation through the continued pursuit of their customary land use practices. Among East African pastoralists, there is concern that traditions and cultural values associated with livestock keeping that have enhanced the coexistence of people and wildlife are changing, and being replaced by new livelihoods and values that increase human-wildlife conflict and wildlife loss [126]. Cultural practices that promote healthy ecosystem functioning are better viewed as a suite of activities, values, and relationships that cannot be divorced from socio-political and economic contexts, and importantly, are developed in and with particular landscapes. Workshop participants shared this awareness, and were also perturbed by cultural losses associated with people’s sense of place having negative consequences for human and ecosystem well-being [127], and declining access to and stewardship over resources, which threaten to diminish aspects of identity that are contingent on the continuation of landscape practices.

### 5.4 Implication of co-produced scenarios of future LULCC on meeting SDGs, Tanzania’s Vision 2025 and sustainable land management

The SDGs aim to transform the world by 2030 by addressing environmental, economic, and social components of sustainable development. However, achieving a balance between these components is challenging. For instance, Tanzania has achieved a 5–7% annual economic growth rate over the last decade, yet 29% of its population lives below the basic needs poverty line [105,128]. The SDGs associated with the workshop’s discussions were SDG 1 (no poverty), 2 (zero hunger), 3 (good health and well-being), 8 (decent work and economic growth), 11 (sustainable cities and communities), 13 (climate action) and 15 (life on land). Currently, 15%, 18%, and 14% of the population in Arusha, Manyara, and Kilimanjaro regions, respectively, live below the basic needs poverty line [128] and thus poverty remains a challenge in northern Tanzania. Poverty alleviation occurs simultaneously with SDG 3, SDG 8, and SDG 11: under scenario one, two, and four poverty levels in 2030 are projected to reduce compared to 2018 as growth in infrastructure and markets occur and people become less directly reliant on natural resources for their livelihoods. However, the unsustainable use of natural resources and limited access to markets and infrastructure in scenario three will challenge the achievement of SDG 1. Agricultural expansion in all four scenarios implies that employment in the sector in 2030 will be higher than in 2018. However, ensuring zero hunger by 2030 will be challenging under all scenarios as agricultural technologies will be basic and there will be an imbalance in food distribution between production and consumption zones. Moreover, arable land demand for Tanzania in 2030 under a ‘business as usual scenario’ is projected to be 190,079 km^2^ [84], meaning that our study area will need more land for food production in 2030. Under projected human population growth in 2030, economic and infrastructure growth for the growing population is likely to occur under scenarios one and two which will prioritize adequate infrastructure and market accessibility to improve economic conditions. Scenario two will, however, also prioritize sustainable development and its economy will be lower than for scenario one. Working towards SDG 13 (climate action) and 15 (life on land) goals, heightened environmental awareness in 2030 under scenarios two and four will improve the protection, restoration, and sustainable use of terrestrial natural resources and cultural heritage sites, whereas scenarios one and three will be challenged by degraded land, deforestation, and unplanned land uses.

The 2030 sustainability targets and the time frame of the SDGs is also close to Tanzania’s development blueprint ‘Vision 2025’, which aims to advance economic transformation through industrialization, poverty reduction, and environmental sustainability [104,128]. To achieve the SDGs and the Vision 2025 sustainability targets, northern Tanzanian landscapes should be viewed as multifunctional coupled social-ecological systems whose management should integrate wildlife and cultural heritage conservation with well-being [17,40,129]. By connecting local actions and global challenges, and acknowledging the role of local cultural heritage as significant in driving land use, the UNESCO World Heritage Sites (WHS) and MAB land management approach provides an example of how landscapes can achieve the sustainability targets of national and international development agendas. Across northern Tanzania, the geopark and Lake Manyara have UNESCO-WHS MAB status. If more multiple land use areas within northern Tanzania are included in UNESCO WHS- and MAB-type jurisdictions, the conservation and sustainability of land-use systems will be promoted [33], with the overarching goal being to meet the social, economic and environmental SDGs targets and promoting both sustainable conservation and the improvement of livelihoods across northern Tanzania.

## Conclusion

Scientific and research communities in northern Tanzania are increasingly interested in the current and future challenges around climate change, population growth and land use transitions. The development of generalized LULCC knowledge summaries, generated from diverse sources, communicated to several audiences, can be useful for supporting dialogues to frame issues around sustainable and inclusive development [44,75]. Generalized knowledge, however, poses significant challenges to developing sustainable future development pathways for northern Tanzania because the area is characterized by varying environmental gradients, biodiversity, livelihood strategies, economic development, historical trajectories, and land uses. Practical solutions for addressing the future sustainability of the multifunctional landscapes of northern Tanzania include participatory scenario development that supports local decision-making, social learning, and collective actions to address common objectives. Using the Kesho framework, our study illustrates the importance of facilitating interactions between stakeholders and researchers to assess historical and future LULCC. Our study further shows that, although the Kesho participatory modeling framework has been used in different studies in East Africa to envision land cover change scenarios, its application in northern Tanzania faced some few challenges. Firstly, Kesho outcomes rely on diverse stakeholders to co-produce future LULCC scenarios. Our study optimized the diversity of the stakeholders involved, but this same diversity also presented challenges in the way of engaging all community representatives and professional stakeholders in an equal capacity, due to their divergent ways of interpreting, communicating and understanding human-environmental interactions. To address the challenge, more time was allocated for discussion to ensure stakeholders could reach consensus, or at least mutual levels of understanding, on various topics. Secondly, it was challenging for stakeholders to relive the past and use that information to project the future principally due to lack of familiarity with participatory scenario development processes. To address the challenge, we engaged the stakeholders before the workshop, discussed with them the objectives of the workshop and sent them some literature about Kesho. Thirdly, an advertised benefit of attending the workshop in Karatu was the training and exposure gained by attendees on methods of participatory scenario development. However, the full process of integrating stakeholders’ insights with spatial modelling required expert knowledge and modelling skills that could really best be provided by attending training on the Kesho framework, making it difficult for many participants in the workshop to replicate this method without further instruction. Finally, though our study has produced scenarios of future LULCC, the scenarios do not predict what is going to happen in 2030 but provide four alternative and plausible trajectories of future environmental change. We conclude that different management strategies for protecting wildlife and cultural heritage sites are key determinants of future LULCC under increasingly divergent climates and socioeconomic factors. Effective management strategies for future landscapes in northern Tanzania should involve institutions and the public to promote sustainable development of communities, and effective management of natural resources and cultural heritage.

## Supporting information

S1 Table Stakeholder composition at the Karatu workshop in northern Tanzania. (DOCX)

S2 Table Spatial data layers used to indicate the spatial distribution of the factors that will drive land cover change in 2030 and where land cover change is likely to (or not to) occur. (DOCX)

S3 Table Protected areas land cover area (km2) for shrubs (20), herbaceous vegetation (30), agriculture (40), sparse vegetation (60), closed forest evergreen broad leaf (112), closed forest deciduous broadleaf (114) open forest evergreen broad leaf (122) open forest deciduous broadleaf (124) at the baseline map of 2018 and under scenario one (developed economy with degraded land), two (developed economy with healthy land), three (developing economy with degraded land) and four (developing economy with healthy land) in 2030. (DOCX)

S1 Text Future land demand estimate. (DOCX)

S2 Text Karatu workshop stakeholder’s scenario narratives of LULCC in northern Tanzania in 2030. (DOCX)
