# Supplementary material for: Integrating stakeholders’ perspectives and spatial modelling to develop scenarios of future land use and land cover change in northern Tanzania
Source: PLoS One. 2021 Feb 12;16(2):e0245516. doi: 10.1371/journal.pone.0245516 (PMC7880460; doi:10.1371/journal.pone.0245516)
Supplement: S1 Table — (DOCX) [file pone.0245516.s001.docx]

**S1 Table.** Stakeholder composition at the Karatu workshop in northern Tanzania

| **Stakeholder composition at the Karatu workshop in northern Tanzania** | **Percent** |
| --- | --- |
| Community (pastoralists, farmers, religious institute) | 25% |
| District government officers (tourism, road engineer, environmental scientist) | 25% |
| Academic researchers (ecologists) | 15% |
| Parastatal officers (social scientists, environmental scientists) | 15% |
| Regional government officers (geologist, museum curator) | 10% |
| Conservation-based and community-based nongovernment organisations | 5% |
| Media | 5% |
| **Total** | **100%** |
