# Supplementary material for: Integrating stakeholders’ perspectives and spatial modelling to develop scenarios of future land use and land cover change in northern Tanzania
Source: PLoS One. 2021 Feb 12;16(2):e0245516. doi: 10.1371/journal.pone.0245516 (PMC7880460; doi:10.1371/journal.pone.0245516)
Supplement: S2 Table — (DOCX) [file pone.0245516.s002.docx]

**S2 Table.** Spatial data layers used to indicate the spatial distribution of the factors that will drive land cover change in 2030 and where land cover change is likely to (or not to) occur.

| **Input products and datasets** | **Derived datasets** | **Data source and availability** |
| --- | --- | --- |
| Land cover - twelve land cover categories; land cover area (km^2^); gains and losses in area of land cover categories from 2018 to 2030.  Spatial scale – 100m | Land cover in 2018 and 2030 | Buchhorn M, Smets B, Bertels L, Lesiv M, Tsendbazar NE, Herold M, Fritz S. Copernicus Global Land Service: Land Cover 100m: Epoch 2018: Globe. Version V2. 0.2. 2019. [cited 2020 Apr 20]. Available from: <https://land.copernicus.eu/global/products/lc> |
| Land cover - Euclidean distance (m) of a grid to the nearest cultivated land cover grid in 2018.  Spatial scale – 100m | Distance from cultivated areas | Buchhorn M, Smets B, Bertels L, Lesiv M, Tsendbazar NE, Herold M, Fritz S. Copernicus Global Land Service: Land Cover 100m: Epoch 2018: Globe. Version V2. 0.2. 2019. [cited 2020 Apr 20]. Available from: <https://land.copernicus.eu/global/products/lc> |
| Land cover - Euclidean distance (m) of a grid to the nearest built-up land cover grid in 2018.  Spatial scale – 100m | Distance from built-up areas | Buchhorn M, Smets B, Bertels L, Lesiv M, Tsendbazar NE, Herold M, Fritz S. Copernicus Global Land Service: Land Cover 100m: Epoch 2018: Globe. Version V2. 0.2. 2019. [cited 2020 Apr 20]. Available from: <https://land.copernicus.eu/global/products/lc> |
| SRTM 90m Digital Elevation Database v4.1 - (elevation (m) and slope (degrees) of areas that will be suitable (or not) for agriculture in 2030.  Spatial scale – 90m | Elevation and slope | Jarvis A, Reuter HI, Nelson A, Guevara E. Hole-filled SRTM for the globe Version 4. 2008. [cited 2020 Apr 20]. Available from: <https://cgiarcsi.community/data/srtm-90m-digital-elevation-database-v4-1/> |
| AfriPop (Number of people in Africa per grid square, with national totals adjusted to match UN population division estimates (http://esa.un.org/wpp/).  Spatial scale – 100m | Projected human population in 2030 | WorldPop (www.worldpop.org - School of Geography and Environmental Science, University of Southampton). Alpha version 2010 estimates of numbers of people per grid square, with national totals adjusted to match UN population division estimates (<http://esa.un.org/wpp/>). 2013. [cited 2020 Apr 20]. Available from: <https://www.worldpop.org/doi/10.5258/SOTON/WP00078> |
| World Database of Protected Areas -(location of Game Controlled Areas (GCAs), Wildlife Management Areas (WMAs), Forest Reserves, National Parks, and protected Open Areas; conservation status of Protected Area; area coverage (km2) of Protected Areas in 2018.  Spatial scale: Global | Protected Areas | IUCN and UNEP-WCMC. The World Database on Protected Areas (WDPA). Cambridge, UK. 2016. [cited 2020 Apr 21]. Available from:<https://www.protectedplanet.net/> |
| World Database of Protected Areas - Euclidean distance (m) of a grid to the boundary of the nearest Protected Area.  Spatial scale: Global | Proximity to/inside Protected Area boundary; | Same as above. |
| Global roads open data access (gROADSv1) - location of roads; Euclidean distance of a grid to the nearest grid with an all-weather road in 2018.  Spatial scale: Global | Distance from all-weather roads | Center for International Earth Science Information Network - CIESIN - Columbia University, and Information Technology Outreach Services - ITOS - University of Georgia. Global Roads Open Access Data Set, Version 1 (gROADSv1). Palisades, NY: NASA Socioeconomic Data and Applications Center (SEDAC). 2013. [cited 2020 Apr 21]. Available from: <https://sedac.ciesin.columbia.edu/data/set/groads-global-roads-open-access-v1> |
| Mines and minerals occurrences - location of mines for metals, industrial minerals, gemstones; Euclidean distance of a grid to the nearest grid with an active mine.  Spatial scale: National | Distance from mining sites | Geological map of Tanzania. 2020 [cited 2020 Apr 21]. Available from: <http://www.gmis-tanzania.com/> |
| Crop suitability - likelihood of a crop to grow in a particular place considering the mean annual rainfall, pH, lithology, physiography, mean annual temperature, altitude, agro-ecological zone of the place and the growing period of the crop.  Spatial scale: National | Projected (2030) crop suitability | Ministry of Agriculture Training Institute - Mlingano (United Republic of Tanzania). 2020. [cited 2020 Apr 20]. Available from: <https://www.kilimo.go.tz/index.php/en/institutes/view/ministry-of-agriculture-training-institute-mlingano-tanga> |
| [Gridded Livestock of the World (GLW)](https://dataverse.harvard.edu/dataverse/glw) v2.0 - Production systems (livestock units of cattle, goats and sheep for northern Tanzania.  Spatial scale: Global | Projected livestock grazing impact | Robinson TP, Wint GW, Conchedda G, Van Boeckel TP, Ercoli V, Palamara E, Cinardi G, D'Aietti L, Hay SI, Gilbert M. Mapping the global distribution of livestock. PloS One. 2014;9(5): e96084. [cited 2020 Apr 21]. Available from: <http://www.fao.org/livestock-systems/en/> |
| FAO future agriculture land demand (hectares) for Tanzania in 2030.  Spatial scale: National | Projected (2030) land demand (ha) in northern Tanzania under the ‘Business as usual, ‘Stratified Societies’ and ‘Towards Sustainability’ FAO scenarios. | Food Agriculture Organization (FAO).. The future of food and agriculture – Alternative pathways to 2050. Rome. 2018. [cited 2020 Apr 19]. Available from: <http://www.fao.org/global-perspectives-studies/food-agriculture-projections-to-2050/en/> |
| FAO future livestock units demand (1000 heads) for Tanzania.  Spatial scale: National | Projected (2030) land demand (1000 heads) in northern Tanzania under the ‘Business as usual, ‘Stratified Societies’ and ‘Towards Sustainability’ FAO scenarios. | Same as above |
| Administrative boundaries (location of districts and regions in northern Tanzania.  Spatial scale: National | Administrative districts in northern Tanzania | Tanzania National Bureau of Statistics. 2020. [cited 2020 Apr 20]. Available from: http://www.nbs.go.tz/ |
| Location and land cover of cultural heritage sites.  Spatial scale: Local |  | Ngorongoro Conservation Area Authority (NCAA) management. |
