# Supplementary material for: Integrating stakeholders’ perspectives and spatial modelling to develop scenarios of future land use and land cover change in northern Tanzania
Source: PLoS One. 2021 Feb 12;16(2):e0245516. doi: 10.1371/journal.pone.0245516 (PMC7880460; doi:10.1371/journal.pone.0245516)
Supplement: S3 Table — (DOCX) [file pone.0245516.s003.docx]

**S3 Table.** Protected Areas land cover area (km^2^) for shrubs (20), herbaceous vegetation (30), agriculture (40), built-up (50), sparse vegetation (60), closed forest evergreen broad leaf (112), closed forest deciduous broadleaf (114) open forest evergreen broad leaf (122) open forest deciduous broadleaf (124) at the baseline map of 2018 and under scenario one (developed economy with degraded land), two (developed economy with healthy land), three (developing economy with degraded land) and four (developing economy with healthy land) in 2030.

| **Land cover category** | **Land cover 2018 (km^2^)** | **Land cover 2030 (km^2^)** | | | |
| --- | --- | --- | --- | --- | --- |
|  |  | **Scenario one** | **Scenario two** | **Scenario three** | **Scenario four** |
| **Burko Open Area** | | | | | |
| 20 | 106.32 | 120.59 | 118.12 | 152.39 | 120.49 |
| 30 | 579.69 | 494.03 | 527.81 | 551.11 | 553.50 |
| 40 | 131.46 | 223.95 | 187.39 | 162.03 | 159.87 |
| 50 | 0.61 | 0.79 | 0.81 | 0.61 | 0.62 |
| 60 | 0.47 | 0.47 | 0.47 | 0.47 | 0.47 |
| 112 | 15.29 | 15.10 | 15.17 | 14.84 | 15.13 |
| 114 | 19.48 | 14.75 | 16.32 | 9.68 | 16.15 |
| 124 | 84.06 | 67.71 | 71.31 | 46.25 | 71.15 |
| **Burunge WMA** | | | | | |
| 20 | 41.77 | 36.26 | 39.32 | 53.05 | 43.07 |
| 30 | 109.29 | 83.79 | 93.84 | 100.64 | 101.26 |
| 40 | 245.93 | 284.79 | 269.55 | 259.20 | 258.12 |
| 60 | 0.07 | 0.29 | 0.07 | 0.30 | 0.13 |
| 112 | 82.74 | 78.53 | 79.70 | 75.31 | 80.06 |
| 114 | 5.21 | 4.29 | 4.50 | 3.19 | 4.57 |
| 112 | 0.53 | 0.35 | 0.40 | 0.23 | 0.45 |
| 124 | 12.04 | 8.70 | 9.78 | 6.07 | 9.99 |
| **Enduimet WMA** | | | | | |
| 20 | 675.35 | 680.44 | 680.78 | 711.15 | 685.21 |
| 30 | 1387.02 | 1384.06 | 1385.25 | 1385.99 | 1386.25 |
| 40 | 383.11 | 402.07 | 394.75 | 389.55 | 388.91 |
| 50 | 0.75 | 0.79 | 0.79 | 0.79 | 0.79 |
| 60 | 16.42 | 16.42 | 16.42 | 16.42 | 16.42 |
| 112 | 319.36 | 317.27 | 317.87 | 315.79 | 318.01 |
| 114 | 129.37 | 125.55 | 126.63 | 122.46 | 126.92 |
| 122 | 0.13 | 0.12 | 0.12 | 0.11 | 0.12 |
| 124 | 259.10 | 243.91 | 248.05 | 228.45 | 248.00 |
| **Lake Natron GCA** | | | | | |
| 20 | 2461.89 | 2127.92 | 2262.64 | 2381.00 | 2364.65 |
| 30 | 4652.71 | 4232.29 | 4401.62 | 4511.70 | 4523.48 |
| 40 | 472.04 | 1371.74 | 1012.80 | 773.53 | 750.74 |
| 50 | 2.12 | 2.12 | 2.12 | 2.12 | 2.12 |
| 60 | 16.33 | 16.33 | 16.33 | 16.33 | 16.33 |
| 112 | 133.88 | 132.60 | 133.22 | 133.41 | 133.51 |
| 114 | 224.13 | 203.82 | 211.17 | 214.34 | 216.72 |
| 122 | 0.31 | 0.31 | 0.31 | 0.31 | 0.31 |
| 124 | 880.83 | 757.12 | 804.05 | 811.50 | 836.39 |
| **Loliondo GCA** | | | | | |
| 20 | 1799.54 | 1532.59 | 1684.09 | 2064.35 | 1808.57 |
| 30 | 4382.69 | 3819.69 | 4044.62 | 4193.03 | 4208.80 |
| 40 | 257.60 | 1905.86 | 1245.56 | 811.75 | 768.06 |
| 50 | 0.71 | 0.71 | 0.71 | 0.71 | 0.71 |
| 60 | 7.50 | 7.50 | 7.50 | 7.50 | 7.50 |
| 112 | 2.99 | 1.45 | 1.88 | 1.20 | 2.21 |
| 114 | 124.54 | 65.32 | 83.95 | 70.68 | 97.68 |
| 122 | 0.01 | 0.01 | 0.01 | 0.01 | 0.01 |
| 124 | 1675.11 | 917.92 | 1182.66 | 1101.58 | 1357.22 |
| **Lolkisale GCA** | | | | | |
| 20 | 171.18 | 80.17 | 117.42 | 143.42 | 143.91 |
| 30 | 253.82 | 117.10 | 172.77 | 208.38 | 211.91 |
| 40 | 373.81 | 609.75 | 513.79 | 452.36 | 445.99 |
| 124 | 12.39 | 4.93 | 7.68 | 7.30 | 9.56 |
| **Meru Forest Plantation** | | | | | |
| 20 | 2.94 | 28.87 | 27.57 | 85.41 | 30.90 |
| 30 | 11.69 | 11.62 | 11.67 | 11.67 | 11.65 |
| 40 | 50.84 | 85.94 | 71.34 | 63.93 | 62.20 |
| 50 | 4.38 | 15.54 | 12.83 | 6.30 | 6.45 |
| 112 | 66.63 | 42.54 | 48.37 | 33.09 | 52.85 |
| 114 | 51.48 | 25.85 | 33.36 | 19.79 | 37.42 |
| 122 | 0.05 | 0.03 | 0.03 | 0.01 | 0.03 |
| 124 | 46.64 | 24.02 | 29.49 | 14.26 | 33.04 |
| **Monduli Juu Open Area** | | | | | |
| 20 | 76.05 | 93.82 | 92.69 | 142.07 | 96.85 |
| 30 | 825.18 | 635.57 | 712.48 | 762.05 | 767.94 |
| 40 | 277.55 | 490.16 | 404.16 | 348.85 | 342.61 |
| 50 | 2.09 | 6.91 | 5.46 | 2.92 | 2.79 |
| 60 | 4.38 | 4.48 | 4.38 | 4.44 | 4.41 |
| 112 | 70.27 | 63.23 | 64.97 | 61.04 | 66.20 |
| 114 | 34.82 | 22.18 | 25.87 | 17.84 | 27.40 |
| 122 | 1.04 | 1.03 | 1.02 | 1.02 | 1.03 |
| 124 | 86.19 | 60.19 | 66.54 | 37.36 | 68.34 |
| **Mto wa Mbu GCA** | | | | | |
| 20 | 229.46 | 244.19 | 241.17 | 263.79 | 240.84 |
| 30 | 1321.00 | 1320.64 | 1320.79 | 1320.90 | 1320.94 |
| 40 | 386.75 | 385.59 | 385.96 | 386.51 | 386.52 |
| 50 | 1.75 | 3.98 | 3.21 | 2.18 | 2.12 |
| 60 | 6.37 | 6.43 | 6.37 | 6.43 | 6.38 |
| 90 | 0.36 | 0.36 | 0.36 | 0.36 | 0.36 |
| 112 | 115.82 | 115.26 | 115.46 | 114.73 | 115.41 |
| 114 | 85.92 | 84.86 | 85.21 | 83.48 | 85.17 |
| 122 | 0.35 | 0.31 | 0.33 | 0.16 | 0.27 |
| 124 | 147.34 | 133.54 | 136.29 | 116.65 | 137.13 |
| **Simanjiro GCA** | | | | | |
| 20 | 366.75 | 276.30 | 314.55 | 353.71 | 343.63 |
| 30 | 420.65 | 276.61 | 336.80 | 372.63 | 376.59 |
| 40 | 550.95 | 804.32 | 700.37 | 634.89 | 628.16 |
| 50 | 0.18 | 0.61 | 0.49 | 0.21 | 0.24 |
| 112 | 0.56 | 0.16 | 0.30 | 0.11 | 0.36 |
| 114 | 1.65 | 0.59 | 0.89 | 0.40 | 1.07 |
| 124 | 58.46 | 41.35 | 46.45 | 38.40 | 49.56 |
