# Supplementary material for: Integrating stakeholders’ perspectives and spatial modelling to develop scenarios of future land use and land cover change in northern Tanzania
Source: PLoS One. 2021 Feb 12;16(2):e0245516. doi: 10.1371/journal.pone.0245516 (PMC7880460; doi:10.1371/journal.pone.0245516)
Supplement: S1 Text — (DOCX) [file pone.0245516.s004.docx]

**S1 Text**

**Future land demand estimate**

Based on an annual forest loss of 1% in Tanzania from 1990 to 2010 (Rosa et al., 2018), we used a fixed forest loss rate of 1.0% per annum to estimate the amount of forest that will be lost in our study area from 2018 to 2030.

To estimate agriculture land demand in 2030, we used the FAO, 2018, scenarios of crop production (hectares) for arable land in Tanzania in 2030. The FAO data calculated land demand (ha) in 2012, 2030 and 2050 under business as usual, stratified societies and towards sustainability scenarios. From the FAO data, we excluded the crops that do not grow in our study area, then converted the 2030 crop production land demand data from hectares to square kilometres and estimated the land demand required for our study area using data from the FAO scenarios. We used agriculture production land demand data from the ‘stratified societies’ scenario to estimate agriculture production land demand for our scenario one (developed economy with degraded land) and three (developing economy with degraded land). For estimating agriculture production land demand for our scenario two (developed economy with healthy land), we used estimates from the ‘towards sustainability’ FAO scenario and for estimating agriculture production land demand for our scenario three (developing economy with healthy land), we used estimates from the ‘business as usual’ scenario.

Based on stakeholders’ opinions and research, increased livestock activities have increase sparse vegetation and bare ground in our study area (Blake et al., 2018). We used projected livestock units data for Tanzania in 2030 from the FAO (FAO, 2018), to estimate the impact on projected livestock units on sparse vegetation. We related current livestock units supported in our study area with the amount of land currently categorised as sparsely vegetated, then using livestock units projections for 2030, we estimate the amount of land that is likely to be sparsely vegetated when supporting the livestock units projected for 2030. Like the agriculture production FAO projections, the FAO livestock units projections were also categorised under the business as usual, stratified societies and towards sustainability scenarios which we related to our scenarios the same way as we did when estimating agriculture land demand.

Following an annual urbanization growth rate of 5.2% for Tanzania (URT, 2016), we used a fixed rate of 5.2%/yr to estimate the land demand for urban areas in 2030.
