# Supplementary material for: Integrating stakeholders’ perspectives and spatial modelling to develop scenarios of future land use and land cover change in northern Tanzania
Source: PLoS One. 2021 Feb 12;16(2):e0245516. doi: 10.1371/journal.pone.0245516 (PMC7880460; doi:10.1371/journal.pone.0245516)
Supplement: S2 Text — (DOCX) [file pone.0245516.s005.docx]

**S2 Text.** Karatu workshop stakeholder’ scenario narratives of LULCC in northern Tanzania in 2030

**Scenario one - Developed economy with degraded land**

Scenario one emphasizes the role of better-developed and adequate infrastructure and accessible markets in shaping economic, social, and environmental patterns in northern Tanzania when local land health is ignored. Under this scenario, weak governance, political interference or lack of political commitment to utilize participatory stakeholder engagement to develop land-use plans will lead to the development of ‘top-down’ land-use plans that will not be implemented. The focus of decision makers at government and non-governmental organizations will be on the economic development of northern Tanzania without valuing environmental sustainability. Decision makers and other land-use stakeholders will view economic development as a pathway to providing decent livelihoods, higher education levels, better health provision, and industrialization. Natural resources will be exploited as this scenario will lead to expansion of agriculture to improve food security of northern Tanzania in wet and fertile areas that are currently not protected, and to efforts to increase the number of jobs provided by the agriculture sector. Infrastructure development will also be a priority and the decision makers will plan to meet urbanization land demand by 2030. Other priorities for this scenario will be to attract local and foreign investors to invest in land-based ventures, investment in education, and diversified alternatives to land-based livelihoods. Challenges under this scenario will include biodiversity loss, deforestation, land degradation, and competing land-uses between agriculture, wildlife conservation and urbanization. In terms of lifestyles, increased urbanization will attract immigrants into northern Tanzania. Most people will also abandon and change their traditional practices in favor of modern lifestyles characterized by high levels of education. This will lead to changes in traditional livelihoods, such as pastoralism and hunting and gathering, in favor of formal employment.

**Scenario two - Developed economy with healthy land**

Scenario two prioritizes the role of good governance structures, better access to markets, highly developed infrastructure, and heightened environmental awareness in improving the socioeconomic status of societies and the environmental integrity of landscapes in northern Tanzania. This scenario will be characterized by planned and regulated urbanization, infrastructure development, and other land uses. The population will be highly educated and involved with environmental, cultural, and biodiversity conservation.

Land use plans in northern Tanzania will be developed through systematic participatory approaches involving local communities, decision makers, non-governmental and governmental institutions. These plans will be implemented, and their implementation will be regularly monitored to ensure all land systems work efficiently depending on changing climatic patterns and the policy formulation process.

Natural resources will be used sustainably because of the government’s will to implement well-developed land use plans. Protected Area boundaries will experience less encroachment. Cultural zones, such as sacred areas where traditional ceremonies have taken place for generations, will also not be encroached by other competing land uses. Livestock herding will still be practiced but livestock numbers, and subsequently their grazing patterns, will be negotiated and regulated by district councils according to available pasture. The goal of this will be to avoid overgrazing and habitat degradation. Agriculture expansion will also be regulated to ensure there is food security, provision of employment opportunities, and avoidance of encroachment into Protected Areas and riverine zones. Sustainable practices will be used in farming to reduce soil erosion and degradation and limit other practices that cause environmental issues and amplify climate change impacts. There will not be high levels of deforestation as most people will not be using fuel wood for energy; rather they will be using gas and renewable energy sources, such as wind and solar power. The key priorities for this scenario will be ensuring food security, protecting wildlife spaces and cultural heritage sites, and increasing socioeconomic development. The key challenge this scenario will face will be balancing infrastructure development, environmental conservation, livestock grazing, and agriculture land uses in northern Tanzania.

**Scenario three - Developing economy with degraded land**

This scenario will be characterized by weak governance, basic infrastructure, lack of environmental consciousness, and complacent communities. Under this scenario, natural resource management policies will either not be implemented or will be poorly implemented and enforced. This will lead to deforestation, exploitation of natural resources, overgrazing, and encroachment of smallholder agriculture in semi-arid areas that do not receive high-priority protection. Deforestation will be driven by excessive use of charcoal, fuel wood, and timber. Overgrazing will reduce dry season pasture and herders will be forced to graze in Protected Areas where grass biomass may be slightly higher than what is available to livestock on their land. This will create livestock-wildlife conflict and will negatively affect community attitudes toward conservation. Lack of political will in establishing and implementing adequate natural resource management plans will further impact wildlife as Protected Areas will not receive proper and timely management interventions for land-use, climate change, and wildlife diseases. These diseases will also be spread to livestock when wildlife and livestock graze together and immunity levels are not monitored. Bush fires will be common as they will be used to control animal pests and diseases as most people will not be able to afford deworming drugs, to pay for tests, or vaccination of their livestock. The general population will lack the expertise of controlling and reducing animal infections when there are disease outbreaks at a public health and governance-level scale, owing to a loss of traditional veterinary and ecological knowledge.

Land use management will equally be unplanned and will lack a participatory approach in its development. Poor agricultural practices, such as burning crop remains, mono-cropping, reduction of fallowing, and farming along riverine areas on the upper watersheds of river networks also characterize this scenario.

Population will increase and will be characterized by low levels of universal primary and secondary education and basic professional skills, with a large pool of unskilled laborers. Local and foreign investors will be discouraged from investing due to low-level skillsets, weak governance institutions, poverty, and unsustainable use of ecosystem services.

Key priorities for this scenario will be to mobilize available natural resources, primarily wildlife and minerals, for revenue generation. Other priorities will be to provide facilities and equipment that can enhance smallholder farming, livestock grazing and other land-based livelihoods. The key challenge for this scenario will be low livestock numbers, poorer quality and inefficient agricultural production, land degradation, and biodiversity loss.

**Scenario four - Developing economy with healthy land**

In this scenario, environmental awareness is key and is combined with good governance structures to minimize the economic effects of basic infrastructure, low market access, and poor economic development. This scenario will have good governance structures, policies, and enforcement that will use participatory approaches to develop and implement land-use plans. Developing the land-use plans will incorporate existing agricultural areas and practices, local community perceptions of wildlife conservation, traditional knowledge, and a high level of sustainability for organizing and developing areas where infrastructure, urban centers, and settlements develop. Infrastructure development, though relatively minor, will be controlled and regulated. Less urbanization will not discourage people from preserving their environment. The population will value and appreciate how their environment supports their livelihoods and cultural traditions.

Low industrialization levels will discourage mechanized agriculture and promote smallholder agriculture that will use the more sustainable aspects of traditional farming practices to promote environmental integrity and promote recognizable social-ecological systems. However, because of the low level of agriculture technology, the agriculture expansion will neither alleviate the food security of northern Tanzania nor provide accelerated economic growth to populations practicing subsistence-level farming in the agricultural sector. There will be low levels of livestock grazing compared to present levels, and the livestock numbers and grazing rotation will be regulated to avoid overgrazing.

The key priorities for this scenario will be to secure wildlife spaces, reduce biodiversity loss, support indigenous knowledge systems and cultural values, and increase tourism, and local and foreign investment into northern Tanzania, and promote agriculture in unprotected but fertile areas. The key challenges for this scenario will be maximizing tourism revenues when infrastructure remains at a lower level of development, relatively low agricultural production, and poorer access to markets for economic growth.
